# Environmentally Relevant Antibiotic Concentrations Exert Stronger Selection Pressure on River Biofilm Resistomes than AMR-Reservoir Effluents

**DOI:** 10.3390/antibiotics13060539

**Published:** 2024-06-10

**Authors:** Gabriela Flores-Vargas, Jordyn Bergsveinson, Darren R. Korber

**Affiliations:** 1 Food and Bioproduct Sciences, University of Saskatchewan, Saskatoon, SK S7N 5A8, Canada; gabriela.flores@usask.ca; 2 Environment and Climate Change Canada, 11 Innovation Blvd., Saskatoon, SK S7N 3H5, Canada; jordyn.broadbent@ec.gc.ca

**Keywords:** reservoir, hotspot, community, biofilm, resistome, mobilome, antibiotics, microbiome

## Abstract

Freshwater environments are primary receiving systems of wastewater and effluents, which carry low concentrations of antibiotics and antimicrobial-resistant (AMR) bacteria and genes. Aquatic microbial communities are thus exposed to environmentally relevant concentrations of antibiotics (ERCA) that presumably influence the acquisition and spread of environmental AMR. Here, we analyzed ERCA exposure with and without the additional presence of municipal wastewater treatment plant effluent (W) and swine manure run-off (M) on aquatic biofilm resistomes. Microscopic analyses revealed decreased taxonomic diversity and biofilm structural integrity, while metagenomic analysis revealed an increased abundance of resistance, virulence, and mobile element-related genes at the highest ERCA exposure levels, with less notable impacts observed when solely exposed to W or M effluents. Microbial function predictions indicated increased gene abundance associated with energy and cell membrane metabolism and heavy metal resistance under ERCA conditions. In silico predictions of increased resistance mechanisms did not correlate with observed phenotypic resistance patterns when whole communities were exposed to antimicrobial susceptibility testing. This reveals important insight into the complexity of whole-community coordination of physical and genetic responses to selective pressures. Lastly, the environmental AMR risk assessment of metagenomic data revealed a higher risk score for biofilms grown at sub-MIC antibiotic conditions.

## 1. Introduction

Worldwide concern over the antimicrobial resistance (AMR) crisis has directed studies to focus on systems where antimicrobial use is high, such as clinical or animal husbandry settings, and/or where antibiotics concentration are elevated (ng/L to mg/L range), such as in wastewater treatment plants (WWTP). These areas are commonly referred to as AMR reservoirs or ‘hotspots’, as there is evidence indicating elevated occurrences of antibiotic-resistant bacteria (ARB) and antibiotic resistance genes (ARGs), underscoring the importance of AMR monitoring campaigns in these priority areas and developing strategies to continue reducing antimicrobial use [1,2,3,4].

Freshwater aquatic environments are the primary receiving systems of WWTP effluent discharge and agricultural run-off, where pseudo steady-state exposure to “low-levels” or sub-minimum inhibitory concentrations (sub-MICs) of antibiotics (~μg/L range) [5] also putatively supports antibiotic resistance in environmental matrices via selective pressure. A great number of measured environmental antibiotic concentrations fall within the range thought to be above the minimal selective concentrations (MSC; the lowest concentration of antimicrobial agent that allows for selection of resistant bacteria without inhibiting the growth of susceptible bacteria), yet are estimated to range between 1/4 and 1/230 of the MIC for singular isolates or pathogens [6,7]. Recent single-species studies support the notion that resistance acquisition can occur at sub-MICs of antibiotics [8,9,10].

Previous work on riverine biofilm communities, which are naturally occurring communities critical for ecological and food web processes in aquatic environments [11,12,13], further supports the premise that exposure to sub-MIC, or environmentally relevant concentrations of antibiotics (ERCA), directly influences the microbiome, ARGs, virulome (VFGs: virulence factor genes), and physical structure of aquatic microbial communities.

The environmental dispersal of antibiotic residues via run-off events is often accompanied by the dispersion of ARBs and ARGs from various hotspots [13,14,15]. The presence of ARBs/ARGs in WWTP has been found to favor the development of multidrug-resistant bacteria [16], which may pose a further risk to human health [17]. However, there is ongoing debate on whether inputs from AMR reservoirs into the environment specifically facilitate the transfer of ARGs and mobile genetic elements (MGE) to other organisms through horizontal gene transfer (HGT). Hutinel et al. demonstrated that while municipal wastewater did not impact HGT rate, hospital wastewater (carrying higher antibiotic levels) did [18], though notably, the MGE profile from both effluents consisted mainly of plasmids from the IncN group. This would suggest that the physical presence of ARB/ARGs in the absence of selective concentrations of antibiotics is not enough to significantly impact the development of AMR in downstream systems. However, this study only reported HGT events in a recipient *Escherichia coli* strain exposed to bacterial donor communities, which although informative, is limited in representing the reality of complex environmental communities and their cell–cell dynamics. Thus, the direct assessment of various AMR reservoir sources (i.e., agriculture run-offs and WWTP effluents), and the combined influence of sub-MIC or ERCA on the resistome of environmental microbial communities, requires further exploration.

Aquatic biofilm communities present an ideal platform to investigate the impacts of AMR-reservoir environmental contamination. These communities are typically high-diversity consortia and are prevalent in most freshwater aquatic environments. Their lifestyle and physical properties, such as the intimate juxtaposition of member cells and the production of extracellular polymeric substances (EPS), help to limit the diffusion of surrounding chemical stressors such as antibiotics. These protective, physical properties also promote the further adoption of tolerance mechanisms which include cell–cell communication (quorum sensing) and facilitate environmental HGT events [19]. Further, these structural properties of biofilms provide protection from flow-mediated erosion and deterioration, making freshwater biofilms stable environmental reservoirs for the development and maintenance of AMR-related elements (i.e., ARGs, MGEs, virulence traits, etc.) [20,21,22,23,24].

It has been demonstrated that continuous exposure to AMR-reservoirs and/or ERCA influences the physical compositional structure of microbial biofilm communities [13]. These exposures likely impose selection pressure for AMR, thereby shifting ecosystem diversity [1,13,25,26,27]. To better understand the influence and relationships of distinct types of waste/effluent inputs on MGE abundance and the prevalence of risk-related ARGs/ARBs, this study analyzed aquatic biofilm responses to antibiotics at ERCA with and without exposure to AMR reservoir inocula from animal (local swine farm) or human (urban WWTP) effluent sources. Microscopic, metagenomic and culture-based methods were thus used to determine: (1) whether the presence of antibiotics at ERCA, together with joint exposure to AMR reservoir inputs from WWTP (W) or swine manure (M), enhanced the AMR-related burden by enriching resistome elements (ARGs, MGEs and VFGs) in aquatic biofilms; (2) whether the sole direct input of AMR reservoirs (W or M) influenced the resistome and putative pathogen profiles of environmental biofilms, and (3) whether biofilm microbial communities interacting with these inputs pose a risk to the environment and/or public health.

## 2. Materials and Methods

### 2.1. Microcosm Experimental Design: ERCA and AMR-Reservoir Treatments Exposure

Microcosm experiments employed rotating annular bioreactors (RABs), as previously described [13]. Natural river water from the South Saskatchewan River, Canada (Figure S1), was collected weekly and used as a source of carbon, nutrients, and microbial communities to develop into biofilms. RABs modelling predominant river conditions consisted of a continuous supply of fresh river water (with appropriate treatment additions to maintain a constant antibiotic concentration over the exposure period) at a delivery rate of one reactor volume (500 mL) per day using a multichannel peristaltic pump (Watson Marlow, Wilmington, MA, USA) with a water temperature of 17 ± 2 °C. The RABs system was designed to provide stable environmental conditions while allowing for controlled testing of the biofilm’s response following addition of ERCA and AMR-reservoir inocula. Physicochemical parameters of the South Saskatchewan River have also previously been described [28] where water was collected within city limits, downstream of the city water treatment plant, yet upstream of both the municipal WWTP discharge facility and sources of agricultural discharge.

Biofilms were provided a one-week establishment phase without antibiotics to initiate growth on the surface of the twelve removable polycarbonate strips (1 × 11 cm) within each replicated RAB. A total of 27 RABs were used in parallel for this study, consisting of nine replicated treatments (*n* = 3) over the study period of eight weeks. The eight-week biofilm development period was based on previous observations made on the same river and RAB study system showing that community diversity and biofilm architecture achieved a pseudo-steady state condition by that time [28,29]. Treatments included RABs continuously exposed to two ERCA or sub-minimum inhibitory concentration (sub-MIC) dosages of an antibiotic cocktail consisting of three different antibiotics: S1: a 10-fold dilution of the MIC values for each antibiotic (1/10 sub-MIC), or S2: a 100-fold dilution of the MIC values for each of the 3 antibiotics (1/100 sub-MIC), and then separately exposed to different AMR-reservoir inocula (W: WWTP effluent or M: swine manure waste organic matter).

**Figure 1:**
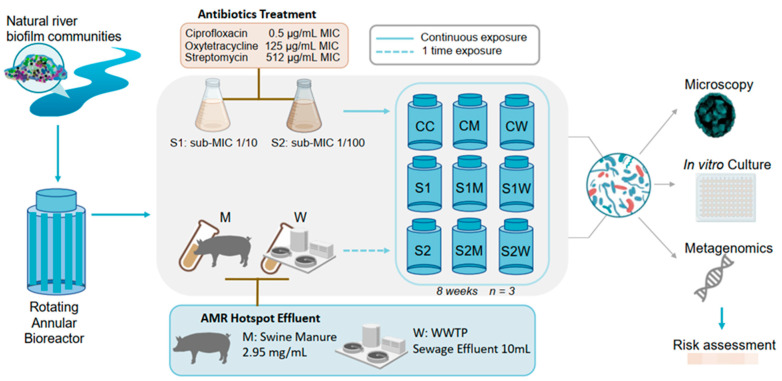
Schematic workflow of the microcosm experimental design used to develop biofilm communities under exposure of nine treatments. RAB: rotating annular bioreactor. MIC: minimum inhibitory concentration. S1: 1/10 sub-MIC, S2: 1/100 sub-MIC. C: river water only. M: swine manure. W: wastewater treatment plant (WWTP).

S1 and S2 sub-MIC antibiotic cocktails were provided by the direct and continuous addition of appropriate dilutions of oxytetracycline, streptomycin and ciprofloxacin at a surface flow velocity of 1.9 km/h (160 rpm) for a period of eight weeks (Figure 1), resulting in μg/L concentrations of the tested drugs within the RAB system. The selection of drugs from three antibiotic drug classes was based on the list of antimicrobial susceptibility surveillance criteria by the WHO, where streptomycin and ciprofloxacin are listed as critically important and oxytetracycline is considered as highly important [30]. Additionally, ciprofloxacin is an antibiotic routinely found in WWTP discharge and oxytetracycline and streptomycin are broad-spectrum antibiotics widely used in Canadian and global livestock operations [1]. The MIC values for the biofilm communities were determined based on breakpoint reports of known pathogen isolates from EUCAST (European Committee on Antimicrobial Testing) and CLSI (Clinical Laboratory Standards Institute) (CLSI, 2018; EUCAST, 2019): ciprofloxacin (MIC 0.5 mg/L), streptomycin (MIC 512 mg/L), and oxytetracycline (MIC 125 mg/L). Concentrations and additional information of the three used antibiotics are available in Supplementary File S2.

For treatments including AMR-reservoir inocula, W and M were added as a one-time “pulse” after completion of the one-week biofilm establishment phase and at the start of the eight-week sub-MIC antibiotic cocktail exposure. After the direct one-time inoculum of M or W, both the reservoir river water delivery (one reactor volume per day) and reactor surface water flow velocity (1.9 km/h; 160 rpm) were turned off for 12 h (to facilitate organism adhesion to the surfaces of the reactor coupons) and thereafter resumed until the experiment’s completion.

The W treatment consisted of WWTP effluent water provided by the City of Saskatoon (Figure S1) and handled as follows; 10 mL volume was filtered (0.2 µm pore-size) as previously described by Guo et al. [21], filters were resuspended in 10 mL of sterile water and vortexed for one minute, then W input was pulse-injected into each RAB.

The M treatment was collected from a local swine operation facility where antibiotics were used [31]. Manure samples stored at −80 °C were thawed, and 85.6 mg was filtered (0.2 µm pore-size). Filters containing the solid retained material were weighted and 30 mL of sterile water was added to meet desired concentration. Based on previous studies [32,33,34], 2.95 mg/mL of manure material was added as inoculum to all RAB systems testing for M effects. Control RABs were operated with river water alone. Figure 1 shows a schematic workflow of the treatments and concentrations used in the microcosm study. At the experiment endpoint, strips were removed from each RAB and directly analyzed for viable biofilm parameters, or frozen and stored at −80 °C for subsequent analyses. All analyses (see below) were conducted on subsamples from randomly selected biofilm strips from each RAB replicate.

### 2.2. Community Structure/Phenotype Using Microscopic Analysis

Coupon pieces of 1 cm^2^ were excised from one randomly selected strip after eight weeks of growth from each RAB and stained for observation using confocal laser scanning microscopy (CLSM, Nikon Eclipse LV 110 DU and C2 camera) with water-immersible lenses (10 × 0.3, 40 × 0.8, 60 × 1.4) (Nikon, Chiyoda, Tokyo, Japan). Biofilm architecture was observed using a three-channel imaging procedure [35,36], including fluorescence from the green (excitation wavelength 488 nm [ex. 488], emission wavelength 522/32 nm [ex. 522/32]), red (ex. 568 nm, em. 605/32 nm) and far-red channels (ex. 647 nm, em. 680/32 nm). Coupons colonized by biofilm communities were directly stained with SYTO9 (Molecular Probes, Eugene, OR, USA) (ex. 488 nm, em. 522–532 nm) to detect nucleic acids of all bacteria. EPS components of the biofilm matrix were visualized using three fluorescent fluor-conjugated lectin-binding dyes at 1 mg/mL concentration: *Triticum vulgaris*-TRITC (TRITC: tetramethyl rhodamine isothiocyanate; ex. 568, em. 605/32) with polymer binding specificity for *N*-acetylglucosamine residues and oligomers (Sigma Chemicals, St. Louis, MI, USA); *Arachis hypogaea*-FITC (FITC: fluorescein isothiocyanate; ex. 485/495, em. 510/600) with polymer binding specificity for galactose and *N*-acetylglucosamine (Sigma Chemicals, St. Louis, MI, USA); and *Canavalia ensiformis*-FITC, also known as “Concanavalin A” (ex. 495/500; em. 495/519), with polymer binding specificity for mannose and glucose residues (Sigma Chemicals, St. Louis, MI, USA). Additionally, chlorophyl autofluorescence from algal and cyanobacteria cells (ex. 647, em. 680/32) was detected in the far-red channel [35]. Five randomly selected locations per coupon were used for Z-stack image scanning with a slice interval of 5 µm (40× lens) and 1 µm (60× lens). CLSM image stacks were collected as previously described by [37] and used for image analysis via ImageJ [38] to define biofilm depth or thickness, biofilm architecture, and biomass of bacteria, EPS and autotrophs.

### 2.3. DNA Extraction, WSG Sequencing and Data Normalization

For each RAB treatment, previously frozen biofilm material from five randomly selected strips were recovered. Biofilm biomass was aseptically obtained using a cell scraper (08-100-241; Fisher Scientific, Pittsburgh, PA, USA) and the collected material centrifuged for 5 min at 9000× *g* to separate the water phase [29,39] and concentrate biofilm material to 300 mg wet-weight. Total DNA was extracted using the Mag-Bind^®^ Universal Pathogen Kit (Omega Bioservices, Norcross, GA, USA) and concentration and yield of DNA was measured with the QuantiFluor dsDNA System on a Quantus Fluorometer (Promega, Madison, WI, USA). Whole genome libraries targeting prokaryotic organisms were constructed using the KAPA Biosystems HyperPlus Kit (Kapa Biosystems, Wilmington, MA, USA) resulting in an average sequencing depth of 36 million reads (Mreads) per sample on an Illumina HiSeq4000/X10 platform.

Paired-end raw reads were quality filtered with Trimmomatic v.0.36 [40] using the following parameters: HEADCROP:10 LEADING:3 TRAILING:3, SLIDINGWINDOW:4:15. Adapters were removed using the TruSeq3 adapter sequence file as reference. All reads were subsampled to a depth of 24 Mreads using the seqkt tool package (https://github.com/lh3/seqtk, accessed on 28 February 2022), as previously described [13,31].

### 2.4. Metagenomic Analysis

The microbiome, VFGs and ARGs profiles were obtained using the CosmosID platform (CosmosID Inc., Rockville, MD, USA). The sensitivity of the CosmosID pipeline was previously tested in a benchmarking analysis comparing metagenomic databases for microbiome and resistome outputs [13]. Trimmed and subsampled FASTQ paired-end reads were merged into a single file per sample and uploaded to the platform for profiling against the platform’s database using default settings for environmental samples to ensure high confidence [31,41,42,43]. For each profile, report files of read count tables were downloaded from CosmosID. Relative abundances were calculated based on the number of specific k-mers and their observed frequency in the sample and then normalized to represent the abundance of each organism and/or gene. Identity of ARGs were corroborated using the output files from CARD (Comprehensive Antibiotic Resistance Database) v.3.0.0 [44] with RGI (Resistance Gene Identifier) application v.6.0.2. Virulome annotations were corroborated via the Virulence Factor Database (VFDB) [45] using default parameters and a cut-off threshold of 90% identity and coverage. Mobilome identity and abundance, focused on plasmids, were estimated using the PlasmidFinder database installed within the ABRicate platform v.1.0.0 [46]. For all AMR-related elements, sequences with ≥95% coverage and ≥85% identity were considered.

Additionally, to offer insight into the safe limits of ERCA used in this study, we assessed the risk for environmental dissemination by using arg_ranker v.3.0.2 [47], a pipeline designed to assess the health risk of ARGs that considers enrichment of human-associated environments, gene mobility, and the presence of ESKAPE pathogens (ESKAPE: *Enterococcus faecium*, *Staphylococcus aureus*, *Klebsiella pneumoniae*, *Acinetobacter baumannii*, *Pseudomonas aeruginosa*, and *Enterobacter* spp.), yielding a risk framework between rank I (highest risk) and IV (lowest risk). Subsampled and merged paired-end FASTQ samples were used as inputs for the analysis.

### 2.5. Antimicrobial Susceptibility Testing

To support the observed metagenomic annotation of MGEs, an antimicrobial susceptibility testing (AST) approach was used to quantify the resistance capacity and estimate the MIC or the end-point antibiotic concentration of bacterial strains from the biofilm communities grown at ERCA or under the influence of AMR-reservoir inocula exposure. The culture-based method employed the U.S. National Antimicrobial Resistance Monitoring System (NARMS). Sensititre NARMS plates (Thermo Fisher Scientific, Pittsburgh, PA, USA) designed for Gram-negative (YCMV3AGNF) and Gram-positive (YCMV3AGPF) isolates were used for screening bacterial strains within the biofilm community (applied as a mixture of unknown cells; see below) for resistance against 14 and 16 different antimicrobials, respectively. Both Gram-negative and Gram-positive NARMS plates included a concentration gradient for ciprofloxacin (0.015–4 µg/mL) and streptomycin (2–64 µg/mL), which were part of our antibiotic cocktail mix for S1 and S2 treatments.

To inoculate the plates, one random strip from each RAB was aseptically scraped and the collected biofilm slurry was then pooled between replicates (*n* = 3) for each of the nine treatments. The collected biofilm material was sonicated in a water bath (Branson Ultrasonics, Danbury, CT, USA) for 5 min and then adjusted to 0.5 McFarland standard using deionized sterile water. Then, 10 µL of the resultant suspended cells were transferred into 11 mL of cation-adjusted (Ca^2+^ and Mg^2+^) Mueller–Hinton broth (Difco, Detroit, MI, USA). *Escherichia coli* DH5-alpha strain was used as a growth control whereas the negative control consisted of only culture media (no antibiotics). A 50 µL aliquot of biofilm culture material, estimated to equal a cell density of ~1 × 10^5^ CFU/mL, was loaded into each of the 96 wells of the NAMRS plates. Plates were incubated at 27 °C and analyzed at 24 and 48 h post-inoculation.

### 2.6. Statistical Analysis

All statistical analyses were performed in R version 4.2.0 [48]. One-way ANOVA was used when data normality assumptions were met (i.e., microscopic analyses, tested using post-Tukey HSD). For non-parametric data, Kruskal–Wallis was applied with the post-hoc Dunn test for significant multiple comparisons between treatments. All *p*-values were adjusted using the Benjamini–Hochberg method to reduce false positive results [49]. α diversity indices such as species richness (Chao1), Shannon, and Simpson were calculated based on the untransformed gene abundance data. Comparisons between control and treatment-exposed communities were assessed by using taxonomic and gene abundance matrices which were normalized with the *decostand* function. Then, permutational analyses of variance based on the Bray–Curtis dissimilarity index (PERMANOVA, 1000 permutations) were calculated from Hellinger-transformed abundance count tables data using the *vegdist* function of the vegan package v. 2.6.2 [50]. Pairwise comparisons between control and treatments were performed to determine significance (*p* < 0.05). Bray–Curtis distance matrices were used to calculate β diversity between samples. β diversity was visualized with non-metric multidimensional scaling plots (nMDS) to ordinate microbiome and resistome data. Significant dissimilarity of ordination between groups was assessed using the Analysis of Similarities statistic (ANOSIM) in the vegan package.

The Bray–Curtis distance matrices from the nMDS ordination were used for correlation patterns between the microbiome–resistome, and virulome–resistome through the *procrustes* function in the vegan package. The Spearman’s rank correlation via the Mantel test was used to assess the relationships between ordination datasets after treatment exposure. Correlations considered significant (*p* < 0.05) and with a dissimilarity coefficient of >0.75 were visualized via network analysis using Hmisc v.4.7-2 and the circlize package v.0.4.15 [51] in R and Gephi v.0.10.1 software [52]. To screen for significant differences (log^2^-fold changes, *p* < 0.05) in gene abundance (for VFGs, ARGs and functional pathways), the DESeq2 v.1.35 [53] package was used where the *contrast* function was used to extract values per treatment condition; the ggpicrust2 v. 1.7.2 [54] package was used to visualize functional pathways and gene families.

## 3. Results and Discussion

### 3.1. Biofilm Structure and Composition

**Figure 2:**
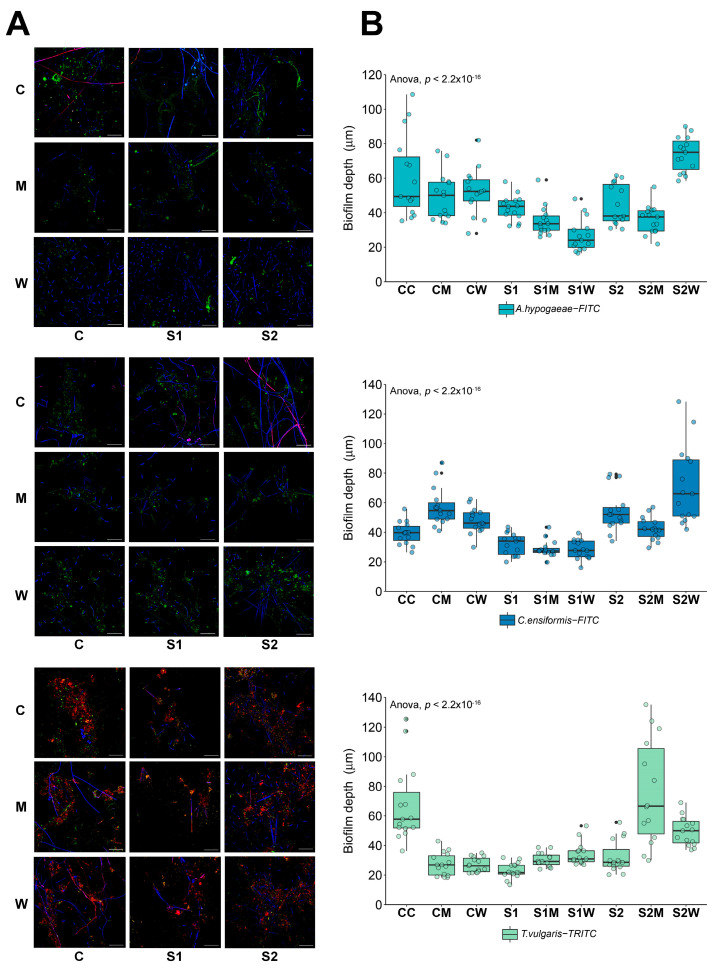
Structural biofilm composition. (**A**) CLSM images stained with *A. hypogaea*-FITC (**top panel**), *C. ensiformis*-FITC (**middle panel**) and *T. vulgaris*-TRITC *+* SYTO9 (**bottom panel**); (**B**) biofilm thickness at different treatments according to fluorescence of lectin-binding specificity to EPS glycoconjugate residues. Mean values are displayed with biological (*n* = 3) and technical (*n* = 5) replicates, edges represent quartile values C = river water only, M = swine manure, W = wastewater treatment plant effluent, S1: 1/10 sub-MIC, S2: 1/100 sub-MIC. Black points indicate the presence of outliers.

The riverine biofilm structure was altered following ERCA exposure (Figure 2) with observed differences in the overall architecture, thickness, and biomass proportion of bacteria, autotrophs, and EPS composition. While pennate diatoms were observed in all samples, cyanobacteria (indicated by magenta fluorescence) were only recorded in control biofilms (Figure 2A).

Pair-wise comparisons of biofilm thickness across samples (Figure S2) revealed significant differences between the control and ERCA-treated biofilms (*p* < 0.001). EPS composition throughout all treatments was primarily dominated by the N-acetylglucosamine (a glycoconjugate of the *T. vulgaris*-TRITC lectin) signal, as previously characterized [13,28,55,56]. Overall, thicker biofilms were recorded for samples grown without ERCA exposure (controls) and S2 (1/100 sub-MIC) antibiotics, whereas thinner biofilms were consistently recorded for the S1, S1M and S1W samples.

The percent biomass abundance of autotroph, bacterial and EPS composition proved significantly different (*p* < 0.001) across treatments (Figures S3 and S4). Autotroph biomass decreased in the S1 treatment (16%); however, biofilm communities amended with M and W increased the percent abundance of autotrophs (27–42.5%), indicating a possible link between the increased nutrients in M and W inocula and the increased autotroph biomass and biofilm thickness in S2, S2M and S2W [57].

The composition and spatial arrangement of biofilm components directly reflects microbial communities’ adaptive responses to external stressors [58]. A more heterogeneous positioning of bacteria within biofilms not only leads to structure variations, but also exposes microorganisms to varying chemical gradients based on their location within the biofilm. Thus, some bacteria could potentially encounter greater selective pressure given the local concentrations of chemicals or agents that surround them [59]. The ERCA and AMR-reservoir exposures in this study proved to alter biofilm structure and, when together at the highest concentrations (S1M and S1W), reduced autotroph and EPS biomass in natural riverine biofilms, which has implications for physical biofilm stability and ecological function.

### 3.2. Microbiome Composition under ERCA Exposure

**Figure 3:**
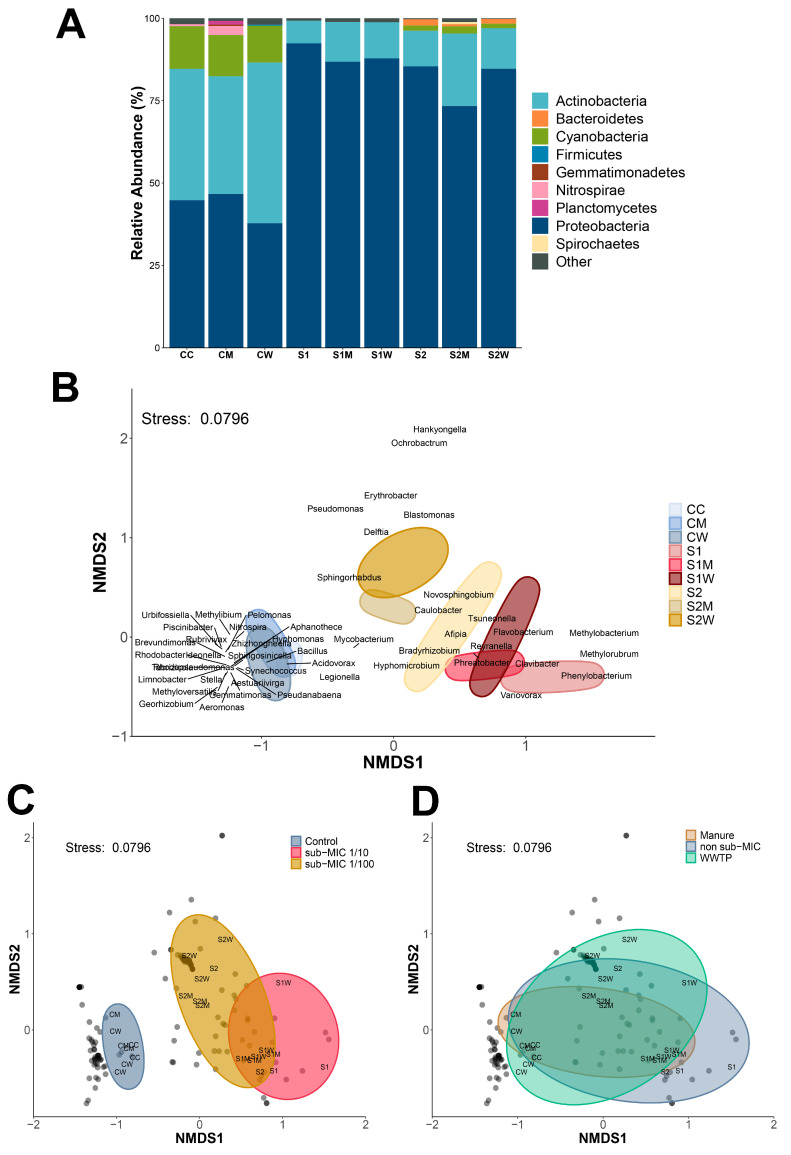
Microbiome profiling. (**A**) Relative abundance of phyla groups and (**B**) nMDS of Bray–Curtis similarities showing β diversity of bacteria composition at the genus level with significant abundance differences across treatments (49 = genera, *p* < 0.05), and nMDS ordination of bacterial β diversity at genus level according to (**C**) AMR-reservoir inocula and (**D**) sub-MIC antibiotic treatments. C = river water only, M = swine manure, W = wastewater treatment plant effluent, S1: 1/10 sub-MIC, S2: 1/100 sub-MIC.

Biofilm exposure to ERCA and AMR-reservoir inocula decreased microbial diversity relative to control biofilms. A total of 15 phyla groups were detected using taxonomic profiling (Figure 3A), with 3 bacterial phyla dominating all treatments including controls. Proteobacteria abundance increased under antibiotic exposure, whereas the abundance of actinobacteria and cyanobacteria decreased relative to control biofilms. The reduction in cyanobacteria was consistent with the reduced autotroph biomass observed in previous observations [13], indicating a deleterious effect of antibiotics on autotrophic biofilm microorganisms. This has potential important implications for energy metabolism and the production of anabolic by-products (i.e., carbohydrates), and thus leads to decreased biofilm functional complexity [24,60,61,62,63]. A total of 25 different bacterial classes were detected in all treatments, of which 13 demonstrated relative abundances higher than 1%, as shown in Figure S5. A total of 166 genera and 267 species were identified across all biofilm samples. Differences in the richness of bacterial species was significant between treatments relative to controls (*p* < 0.001), and significance was also recorded for α diversity indices (Shannon’s, Simpson’s) (Table S1). At the genus level, richness significantly decreased after the addition of antibiotic treatments relative to control biofilms (*p* < 0.001), with the lowest diversity recorded for the S1 condition, and the highest recorded for S2M.

β diversity of bacterial genera demonstrated distinctive separation of control (CC) samples from S1 and S2 antibiotic treatments (Figure 3B), with significant differences across treatments (ANOSIM, R^2^ = 0.802, *p* = 0.0009; Figure 3C). There was no significant difference between AMR-reservoir inocula-treated biofilms and controls (ANOSIM, R^2^ = 0.0256, *p* = 0.982; Figure 3D). Across different treatments, 49 out of 166 genera displayed significant abundance (*p* < 0.05) following nMDS analysis where most genera (28 taxa) were associated with control conditions. Genera associated with S1 (1/10 sub-MIC) dosage treatments include *Clavibacter*, *Flavobacterium*, *Methylorubrum* and *Variovorax*, whereas *Caulobacter* and *Novosphingobium* were aligned with S2 (sub-MIC 1/100) conditions. *Afipia, Bradyrhizobium*, *Cyanobium, Synechococcus, Phreatobacter, Reyranella, Sphingobium, Sphingoharbus* and *Mycobacterium* genera were prevalent throughout all treatments; however, a significant differential abundance of *Mycobacterium* was not recorded even though decreased abundance for this taxon was detected in S1, S2 and S2M treatments. The *Variovorax* genus stood out for its prevalence across the antibiotic-treated samples. *Variovorax* is an ubiquitous genus found in water and soil environments and has been isolated in America, Europe and Asia [64]. This bacterium also demonstrated a correlation to ARGs in the previous analysis of ERCA-treated biofilms [13], suggesting it may be of use as a resistome ‘sentinel’ species for ARG transmission and prevalence.

Bacteria found within natural freshwater biofilms are mostly non-pathogenic; nonetheless, reads classified as putative opportunistic bacterial pathogens were detected in our biofilms: *Aeromonas* spp., *Legionella* spp., *Mycobacterium* spp., *Escherichia* spp., and *P. aeruginosa*, which are all known to persist in aquatic environments [65]. Interestingly, *P. aeruginosa*, a Gram-negative opportunistic pathogen, was detected in samples of CC, CM, CW, S2, S2M and S2W with slightly higher normalized absolute counts in control samples and with slight significant differences in abundances in S2, S2M and S2W (*p* < 0.05).

### 3.3. Resistome Composition at Biofilm Community Level

**Figure 4:**
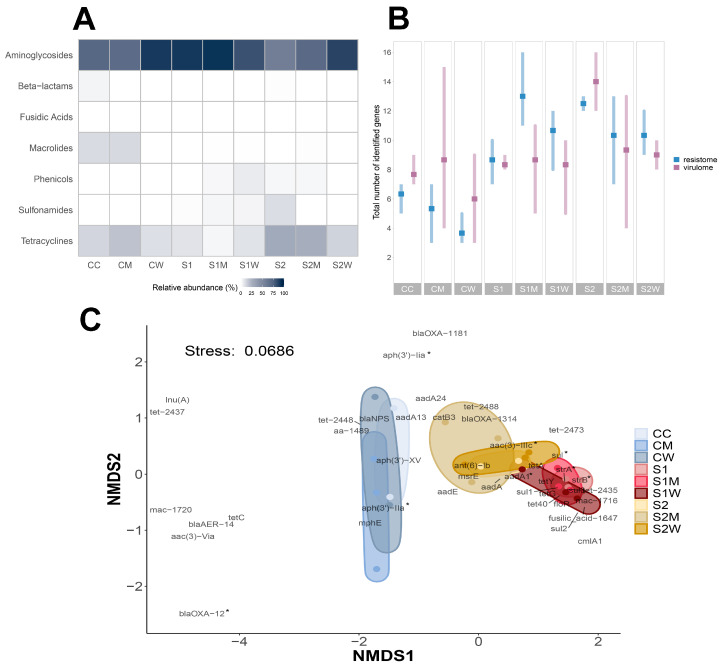
Resistome profiling. (**A**) Relative abundance of total ARGs (42 total identified genes) classified by drug resistance class across biofilm samples; (**B**) richness of resistome and virulome genes; and (**C**) nMDS of Bray–Curtis similarities showing β diversity of total identified ARGs across treatments in biofilm communities. Asterisk (*) indicates ARGs with significant abundance differences across treatments (*p* < 0.05). Ellipse shapes were defined using covariance of each group, and ellipse centroids represents the group mean (*n* = 3). C = river water only, M = swine manure, W = wastewater treatment plant effluent, S1: 1/10 sub-MIC, S2: 1/100 sub-MIC.

Resistome composition analysis identified a total of 263 ARGs across all samples, with 42 different types of ARGs belonging to seven different drug-resistance mechanism classes detected throughout different treatments, including: aminoglycosides, tetracyclines, macrolides, β-lactams, sulfonamides-trimethoprim, phenicol, and fusidic acid. Aminoglycosides were the most abundantly observed ARG class throughout all samples, as shown in Figure 4A, with tetracycline ARGs being the second most abundant genes in antibiotic-treated samples. The prevalence of ARGs was even observed in control samples (CC) and non-ERCA-treated biofilms (CM, CW) such as *strA*, *strB*, *aph(3′)-IIa, aph(3′)-Iia, aadA1*, (aminoglycosides), *tetC* and *tetG* (tetracyclines), indicating the ubiquitous nature of these genes in the aquatic environment with indirect or minor exposures to selective agents (i.e., river water).

The richness of the resistome confirmed that ERCA exposure to 1/10 sub-MIC and 1/100 sub-MIC antibiotic cocktails increased the number of ARGs within biofilm communities. For example, S1M treatment recorded the highest number of resistome genes with 12 ARGs on average (Figure 4B). β diversity of the resistome composition revealed significant differences across ERCA antibiotic-treated samples, but not amongst the AMR-reservoir treated samples (*p* < 0.01; Figure 4C). Data ordination indicated distinctive differences between control and ERCA-treated samples, although overlap between S1W, S1M, S2, and S2W was observed.

**Figure 5:**
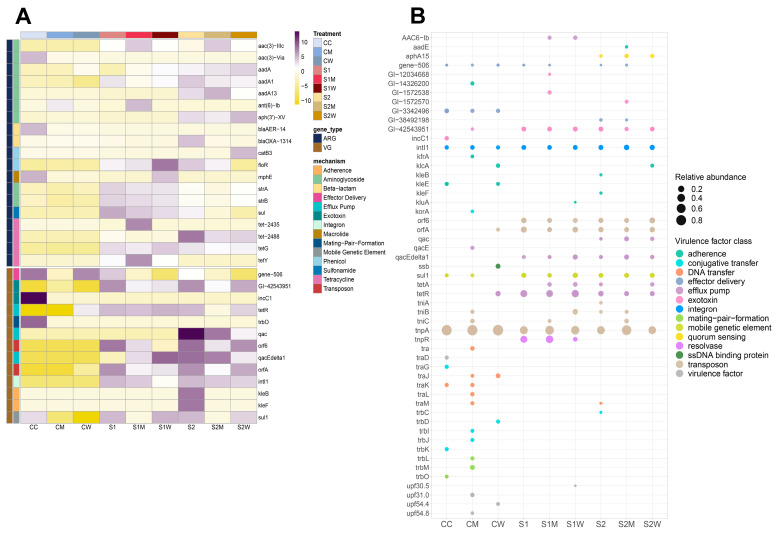
AMR-elements profiling. (**A**) Differential abundance of the resistome of riverine biofilm communities developed under sub-MICs antibiotic and AMR-reservoir exposure. Shown are ARGs with significant fold-changes (*p* < 0.05) between treatments. The displayed normalized abundance is scaled to each gene (Z-score) after rlog-transformed counts; and (**B**) relative abundance (proportional to circle size) of virulence factor genes (53 total identified genes) across biofilm communities under ERCA and AMR-reservoir exposure. Relative abundance indicates the normalized read counts per treatment (*n* = 3). C = river water only, M = swine manure, W = wastewater treatment plant effluent, S1: 1/10 sub-MIC, S2: 1/100 sub-MIC.

Most detected ARGs were associated with antibiotic exposure conditions; however, seven ARGs were associated with control conditions (aminoglycosides: *aadA13*, *aa-1489*, *aph(3′)IIa*, *aph(3′)-XV*; β-lactams: *blaNPS*; macrolide: *mphE*; tetracycline: *tet-2448*). Additionally, differential abundance per treatment was measured using DESeq2, resulting in 19 out of 42 ARGs yielding significant log^2^ fold-change differences (*p* < 0.05) in at least two of the nine treatments (Figure 5A). The sulfonamide ARG *sul1* stood out for showing higher abundance after ERCA (S1, S2) and W (S1W, S2W) exposure, whereas abundance after M (S1M, S2M) exposure was not significant (Figure 5A; Table S2), suggesting *sul1* presence is indicative of ERCA and W conditions.

The gene *floR,* a phenicol ARG prevalent in natural environments [15,66], recorded the highest abundance in the S1W treatment relative to controls. Although *floR* has not yet been identified by the WHO as an ARG known to cause problems in clinical settings, resistome risk assessments by Zhang et al. considered this gene as an ARG with a Rank I score (the highest environmental risk) due to its similarity to known high-risk ARG families [47]. 

### 3.4. Virulome Composition at Biofilm Community Level

The virulome, or the total set of genes that contribute to an organism’s virulence, of riverine biofilm communities, was also analyzed [67]. Virulome gene composition was significantly different across sub-MIC antibiotic conditions (*p* < 0.05). A total of 265 virulence factor genes (VFGs) were estimated across all samples, with 53 different types of VFGs (Figure 5B) allocated into 14 classes of virulence, including: effector delivery, exotoxins, adherence, efflux pumps, quorum sensing, integrons, conjugative transfer, transposons, ssDNA binding proteins, mobile genetic elements, resolvase, DNA transfer, virulence factors (specific mechanism unknown) and mating-pair formation. Based on the VFDB database, VFGs were attributed to 16 pathogenic bacteria: *Bacillus* spp., *Burkholderia pseudomallei*, *Enterobacter aerogenes*, *Enterococcus faecium*, *E. coli*, *Klebsiella pneumoniae*, *Proteus mirabilis*, *P. aeruginosa*, *Pseudomonas putida*, *Pseudomonas* spp., *Salmonella* serovar Infantis, *Salmonella* spp., *Salmonella* serovar Typhimurium, *Serratia marcescens*, *Shigella flexneri* and *Vibrio cholerae.* Out of these bacteria, *Bacillus* spp. and *P. aeruginosa* were also significantly detected in the taxonomic microbiome profiling (Figure 3B).

The *tpnA* transposon gene was not only observed in control samples but was also the most abundant VFG detected throughout all samples with up to 80% of VFGs relative abundance (Figure 5B). Other VFGs were observed only in control samples, as well as CM and CW, such as *tra- trb- upf, korA,* and *ssb,* with virulence mechanisms related to DNA transfer, mating-pair formation, conjugative transfer, and ssDNA binding protein.

The abundance of VFGs conferring mechanisms related to efflux pumps, quorum sensing, resolvase and exotoxins was higher in antibiotic-treated samples relative to control counterparts. For example, *qacEdelta1* (efflux pump) was detected in all antibiotic-treated samples (S1, S1M, S1W, S2, S2M and S2W), *aphA15* (quorum sensing) was detected in S2, S2M and S2W, and *tnpR* (resolvase) was recorded for S1, S1M and S1W. The efflux pump gene (*tetA*) and efflux pump gene regulator (*tetR*) were only found in ERCA-treated biofilms (Figure 5B), but not under control conditions. Based on Zhang et al., we expected a higher abundance of conjugative transfer genes in ERCA-treated samples [68]; however, genes related to conjugative transfer were mostly detected in CC, CM and CW conditions (i.e., *traG, trbD, trbI, trbK, trbJ, korA*).

Ultimately, biofilms exposed to antibiotic treatments (S1, S1M, S1W, S2, S2M and S2W) had greater VFGs richness compared to controls (Figure 5B), with the S2 treatment yielding the highest number of VFGs (average of 14). Virulome composition was also tested for similarity between samples demonstrating significant differences across treatments as well as in samples inoculated with AMR-reservoir input (Table S3). Moreover, twelve VFGs yielded significant abundances between treatments, as shown by asterisks in Figure S6 (ANOSIM, R^2^ = 0.299, *p* < 0.001), including *intI1* and *sul1*.

Normalized abundances of *intI1* and *sul1* genes were greater in antibiotic-treated biofilms; however, both VFGs were prevalent in our study since they were also detected in controls. Integron *intI1* has been proposed as an indicator of AMR due to its linkages with HGT and its capacity of carrying multiple ARGs [69,70]. However, our results suggest caution when observing these two genes, since their presence in river water narrows their potential as specific AMR indicators in aquatic environments.

### 3.5. Mobilome Composition at Biofilm Community Level

The PlasmidFinder database revealed the presence of only two plasmids across samples: IncQ2 of the Inc family and ColRNAI of the Col family. Notably, all samples without ERCA treatments lacked detected MGEs, except for one sample replicate from the CW treatment (Table S4). IncQ2 was consistently observed in S1, S1M, S1W, suggesting that the antibiotic cocktail mix at these ERCA levels promoted the prevalence of these plasmid sequences, given that copies of IncQ2 were not detected in control biofilm communities. IncQ2 was also detected at S2W in two of three replicate samples.

The Inc plasmid family has been shown to carry virulence-associated and AMR genes [71] (such as *strA* and *strB* streptomycin resistance genes) within *Enterobacteriaceae* and *Salmonella enterica* [72]. IncQ plasmids have been recorded in diverse systems outside clinical settings, including pig manure [73]; however, the presence of IncQ2 was not observed in CM or S2M, samples which had exposure to swine manure inocula. IncQ1 plasmids have also been associated with *tetA*, *tetR*, and *sul2* ARGs [74]. The increased relative abundance of *strA*, *strB* and *sul* ARGs observed in the S1, S1M and S1W conditions correlates well with the predicted conjugative mobility annotations. The detection of MGEs in S1-treated samples also supports the assumption that higher concentrations of antibiotics contribute to a higher frequency of HGT events [18], even though this current analysis is limited to only predicting and not confirming the occurrence and mechanism of genetic element mobility.

A response trend for ColRNAI was less clear, as it was present in CW, S1, S1M and S2, yet none of these treatments recorded the presence of ColRNAI in all the corresponding triplicate samples. Additionally, the richness of plasmids estimated using PlasmidFinder were in contrast with the virulence sequences annotated using the VFDB database, as the latter displayed higher relative abundances of conjugative transfer-related genes in control samples (CC), CM and CW, in comparison to the other treatments (Figure 5B). This prediction difference may be due to the fact that PlasmidFinder specializes in detecting replicon sequences as a classifier tool for plasmid grouping [75].

Although the number of detected plasmids was low, our findings reinforce the proposition that ERCA, particularly concentrations equal to or exceeding 1/10 sub-MIC levels (ng/µL), likely promote the abundance and transmission of MGEs. The mobilome response observed in this study indicates that while inputs from M or W sources alone do not promote or enrich for MGEs such as plasmids, the input from ERCA, particularly antibiotics at concentrations equal or higher to their 1/10 sub-MIC, do influence and enrich for MGEs. The broad host range of the detected IncQ raises potential public health concerns due to the potential ease of mobilization in environmental microbiomes and contributes to the prevalence and dissemination of other AMR-elements such as ARGs and VGFs.

### 3.6. Co-Occurrence Patterns of the Biofilm Resistome

**Figure 6:**
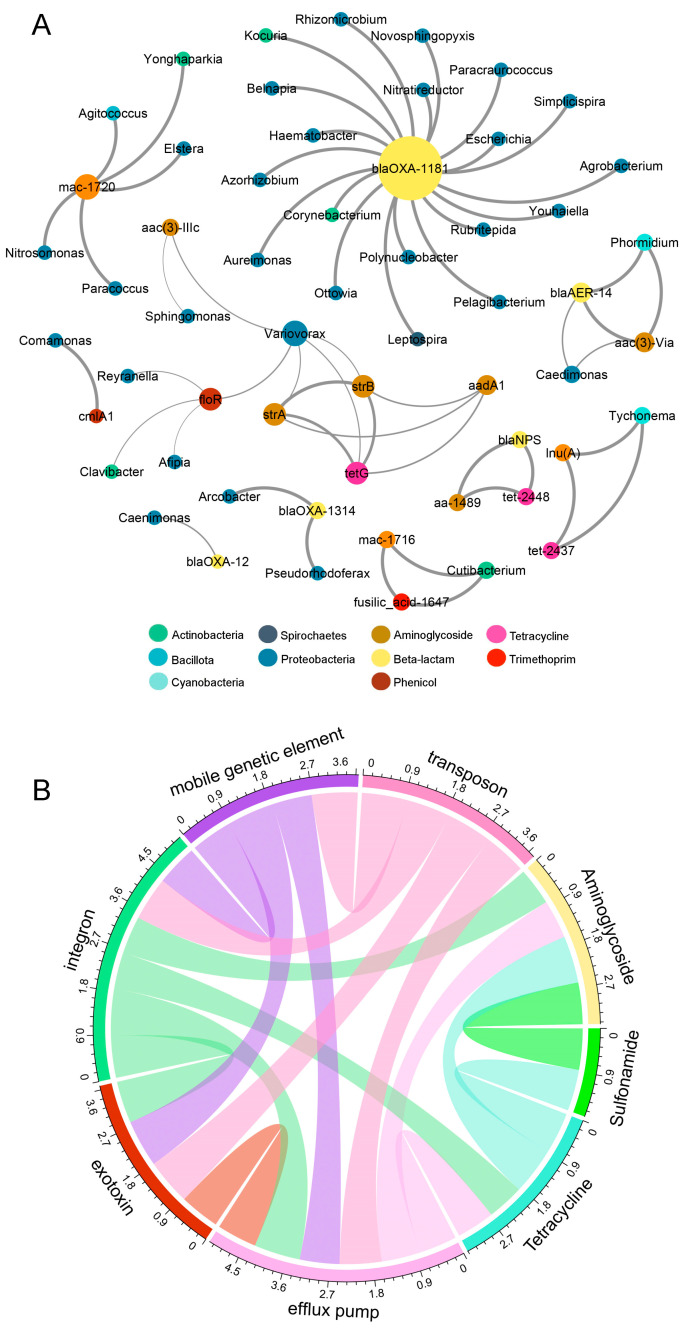
Network analysis of correlations between the (**A**) resistome and microbiome, and the (**B**) resistome and the virulome. Edges (connection lines) denote pair-wise correlations and thickness represent degree of significance (Spearman’s rho > 0.75, *p* < 0.001). Node size is proportional to the number of connections. Outer numbers indicate average degree of co-occurrence (number of edges).

Relationships between the resistome and virulome were investigated to test whether identified ARGs developed under ERCA or AMR-reservoir inocula exposure shared close association to the observed MGE and VFG profiles (Figure 6B). Procrustes analysis demonstrated significant correlation between the microbiome and resistome distance matrices (Mantel r = 0. 520, *p* = 0.001; Procrustes M^2^ = 0.439, r = 0.748, *p* = 0.001; Figure S11). In the network analysis, efflux pumps, MGEs, and exotoxins demonstrated strong relationships with aminoglycoside, tetracycline, and sulfonamide ARGs (Figure 6B). Efflux pumps and integron virulence mechanisms accounted for the greatest number of connections (nodes = 6), a pattern that is also consistent with our metagenomic findings. Efflux pumps are found in almost every bacterial core genome and function in the secretion of substances from the cell, and not solely the removal of antibiotic compounds or other damaging cytoplasmic compounds (e.g., hydrogen ions) [76]. The increased abundance of efflux pump genes in antibiotic-treated samples and close association with integrons further suggests that an increased occurrence of HGT events (i.e., mutations, recombination) within riverine biofilm has been noted [77,78]. Given that ARGs and VFGs occurred even in biofilms grown without added antibiotics, its confirmed that genes encoding for resistance already existed or were previously acquired from natural riverine microbial communities. The challenge of ERCA is sufficient to select for increased resistance-related genes, while the influence of AMR-reservoir inocula (M and W) was less notable on overall resistome character. These findings corroborate the capacity for ERCA to drive resistance selection in aquatic microorganisms, supporting the idea that antibiotics at sub-MIC such as ciprofloxacin, oxytetracycline and streptomycin maintain and potentially disseminate AMR throughout the environment.

### 3.7. Implications to Resistome Risk Assessment in Natural Biofilms

To estimate resistome risk in aquatic biofilms for potential human health risks associated with the dissemination of the ARGs and MGEs present in these natural biofilms, the arg_ranker tool [47] was used. The Rank Risk score (I–IV) is a framework based on the contribution of each ARG Risk Rank as the average abundance of ARGs of a Risk Rank divided by the average abundance of all ARGs [47].

**Figure 7:**
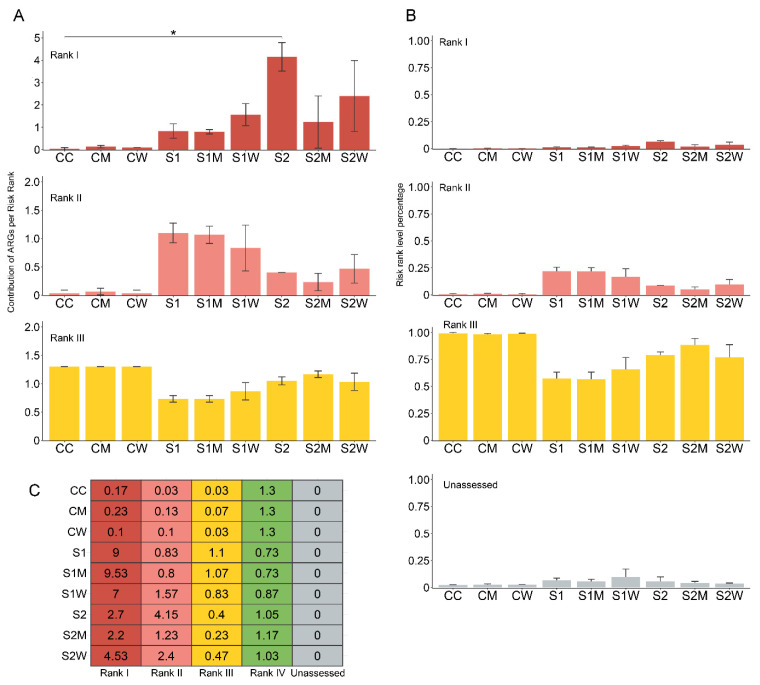
Resistome risk assessment. (**A**) The contribution of ARGs of a Risk Rank based on arg_ranker, (**B**) percentage of ARGs of a Risk Rank, and (**C**) total abundance of ARGs per Rank Risk. Code of contribution is on a scale from Rank I (highest risk to human health) to Rank IV (lowest risk to human health), unassessed refers to ARGs undetected in the metagenome dataset. Data are presented as mean values +/− SD. Asterisk (*) indicates significant difference between treatments (*p* < 0.05). C = river water only, M = swine manure, W = wastewater treatment plant effluent, S1: 1/10 sub-MIC, S2: 1/100 sub-MIC.

The riskiest category of Rank Risk 1 (estimated to carry MGEs recorded in ESKAPE pathogens) was significantly different between the nine treatments (*x*^2^ = 20.869, df = 8, *p*-value = 0.007), specifically between CC vs. S2 (*p* < 0.05) as shown in padj values in Figure 7A. Interestingly, the highest differential abundance of VFGs was also observed for the S2 condition. The Kruskal–Wallis test of Rank Risk II and Risk III also indicated significant differences (*x*^2^ = 22.037, df = 8, *p*-value = 0.004; *x*^2^ = 23.445, df = 8, *p*-value = 0.002, respectively); however, no significant padj results were recorded. No hits were recorded at Rank Risk IV score. Additionally, no Unassessed Risk Values were reported in our data samples, although Unassessed ARG percentages (2.3–10%) were recorded, as shown in Figure 7B.

The majority of ARGs per Risk Rank was encompassed in Risk III across all samples, although the difference was greatest between non-antibiotic-treated (CC, CM, CW = 98%) and 1/10 sub-MIC antibiotic-treated samples (S1, S1M, S1W = 56–65%) (Figure 7B). Risk II results showed a similar pattern to the mobilome richness, where the higher number of plasmids were recorded at S1, S1M and S1W. The results combined with the significantly altered biofilm structural composition, particularly observed at treatments with 1/10 sub-MIC antibiotics, confirm that our antibiotic treatment range of 50–51,200 μg/L exerted the greatest selective effects on river biofilm communities.

### 3.8. NARMS Analysis

To determine whether the constituent members of biofilm communities had greater phenotypic resistance under different treatments, an AST assay using NARMS plates was performed. This was performed with the understanding that the application of NARMS plates is not routine and that each antibiotic treatment exerts a selective pressure to support the growth for only the resistant organisms within the community, and not necessarily all community members.

**Figure 8:**
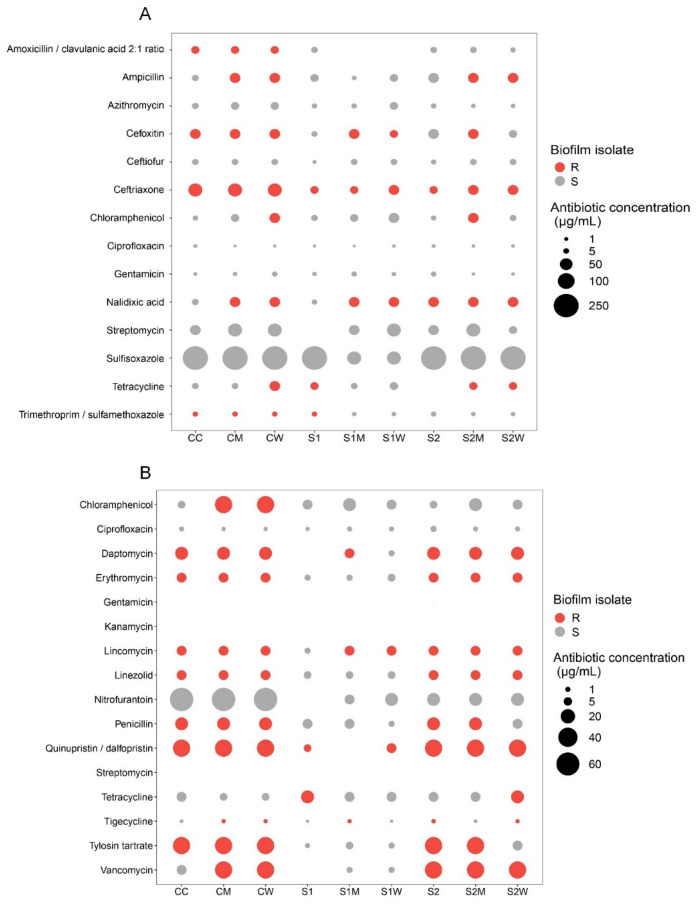
MIC resistance (R) and susceptibility (S) results from NARMS testing for (**A**) Gram-negative and (**B**) Gram-positive bacteria recovered from biofilm inocula grown for 8 weeks under different ERCA treatments (1/10 and 1/100 sub-MIC levels) and AMR-reservoir inoculant supplementation (W and M). C = river water only, M = swine manure, W = wastewater treatment plant effluent, S1: 1/10 sub-MIC, S2: 1/100 sub-MIC.

Both control and all treatment biofilms were resistant to eight antibiotics in plates designated for non-fastidious Gram-negative bacteria (Figure 8A), and eleven antibiotics within plates designated for non-fastidious Gram-positive bacteria (Figure 8B). All samples recorded resistance to ceftriaxone, a third-generation cephalosporin antibiotic, at concentrations higher than the MIC (≥4 µg/mL). Given that the sub-MIC antibiotic cocktail mix used for the microcosm experiments consisted of oxytetracycline, streptomycin and ciprofloxacin, we expected biofilm samples to display some level of resistance to the latter two drugs as they were included in the NARMS plate test. However, no resistance to ciprofloxacin (MIC ≤ 0.5 µg/mL) or streptomycin (MIC ≤ 512 µg/mL) were observed for either Gram-negative or Gram-positive NARMS plates. There was, however, recorded resistance to tetracycline from S1 and S2W samples in both Gram-negative and Gram-positive assays (MIC ≥ 16 µg/mL).

Overall, biofilm inocula from non-ERCA (CC, CM, CW) and 1/100 sub-MIC treatments (S2, S2M, S2W) demonstrated higher resistance in comparison to 1/10 sub-MIC grown samples (S1, S1M, S1W). This observation is contrary to the in silico results which indicated all S1 samples experienced increased gene abundance related to resistance, virulence, and gene transfer. It potentially suggests a complex phenomenon occurring at the community level, whereby the weakened structural stability of the biofilm, as a result of changes in the community composition (i.e., autotrophs vs. heterotrophs) at the highest ERCA, may actually contribute to the increased exposure of vulnerable or sensitive constituent members to the antibiotic compound. Increased phenotypic resistance (at the community level) in very dilute ERCA could thus be the result of the relatively increased physical stability of the biofilm limiting the diffusion and allowing even moderately resistant organisms at the surface of the biofilm to proliferate.

In support of this notion, bacterial species within riverine biofilm communities exhibited increased biodiversity when grown in the absence of ERCA, or at ERCA levels below the 1/10 sub-MIC range. However, there are limitations with the use of culture-based/AST methods for a whole community due to their physical complexity and genetic diversity, and most significantly, because of their specialized nutritive and growth requirements (i.e., many community members simply cannot be grown in pure culture in vitro). The application of AST to biofilm communities does allow insight into whole-community dynamics against antimicrobial challenge vs. the resistance mechanisms of a singular isolate, which is an instance unlikely to occur in natural environments.

Ultimately, this is a major challenge for biofilm-AMR research, given the unstandardized parameters for determining antibiotic susceptibility in multispecies bacterial consortia. Classic antibiotic susceptibility tests such as MIC determined using ECUAST or CLSI were primarily designed for planktonic clinical bacterial isolates, which may not correspond to the necessary concentrations to eradicate their biofilm counterparts, much less for polymicrobial biofilms [76,79].

The results from our NARMS culture-based examination provide a community-level approach for comparing the putative resistances from ARG and MGE occurrences observed from metagenomic data. It is noteworthy that this approach also offers novel opportunities that include the possible isolation and identification of organisms or groups of organisms from complex matrices that can grow within NARMS plates.

### 3.9. Predicted Community Dynamics—In Silico vs. Phenotypic Data

Increased efforts to analyze both environmental (i.e., largely non-pathogenic) bacteria in addition to high priority (pathogenic or clinically isolated) organisms should undoubtedly provide more contextual information on the potential HGT of ARGs, VFGs, and MGEs in natural settings [69]. Based on our microbiome data (Figure 3B), we propose that proteobacteria, and particularly the *Variovorax* species, should be explored as indicators of increased AMR in monitoring campaigns targeting downstream freshwater environments. *Variovorax* specifically may serve as an index of antibiotic or stress tolerance in aquatic environments, as has previously been suggested for *Aeromonas* spp. [80], given that the in silico prediction of *Variovorax*’s abundance has been strongly correlated to antibiotic exposure and ARGs occurrence in freshwater biofilms in this study and previously [13].

It is critical to reflect on the utility of metagenomics and/or environmental omics to reveal or predict ultimate AMR response and potential health outcomes. The antimicrobial and heavy metal concentrations of the river water and W and M AMR-reservoir inputs were also analyzed (for methods see Supplementary File S2), detecting multiple metals and pharmaceuticals, at relatively low concentrations (Tables S5 and S6). Moreover, the metagenomic functional prediction of resistome and stress-response metabolism revealed a higher relative abundance of gene families associated with metal stress response in samples treated with 1/10 sub-MIC antibiotics (Figure S8). Despite the low measured concentrations of these metals, the functional profiling of these genes supports the assertion that the presence of antibiotic selective pressure favors the maintenance of metal resistance genes [33,80].

Multiple lines of analysis herein have demonstrated that higher ERCA result in decreased microbial diversity and increased in silico prevalence of AMR-related elements (resistome, virulome and mobilome), and further alterations in community metabolic function (see Figures S7–S10).

Antibiotic concentrations of this study also had a greater deleterious effect on the structural integrity of the biofilm community. Paradoxically, however, these impacted biofilms were more susceptible to subsequent challenges with antimicrobials than were control or more dilute ERCA-exposed biofilms were.

This highlights the immense challenge of understanding environmental dynamics in the context of AMR transmission and predicting broader impacts for human or animal health. Certainly, AMR surveillance should include and integrate culture-based and metagenomic methods to comprehensively elucidate drivers of AMR dynamics and emergence [81], particularly within the framework of environmental risk assessment (ERA) aiming to quantify the safety limits for AMR acquisition and transmission [79].

The growing concern for the AMR’s ‘silent pandemic’ has encouraged efforts for systematic surveillance of drug resistance. While it is important, yet difficult, to establish standardized environmental resistome reports [70], the risk assessment in this study provides compelling evidence of AMR dissemination within riverine biofilms. Data generated from this research contribute by bridging gaps in the environmental aspect of the One Health approach towards AMR mitigation, as well as assisting stakeholders with guidelines for regulation limits in the monitoring of sub-MIC antibiotics.

## 4. Conclusions

Our results indicate that exposure to ERCA and AMR-reservoir inocula influenced the phenotypes and genotypes of riverine biofilm communities, though not to similar extents. Both ERCA and AMR-reservoir inocula resulted in a greater number of resistome elements relative to untreated biofilms, although ERCA exposure appeared to be a stronger driver of resistance genes. The community approach employed in this study demonstrated that higher ERCA conditions impacted the biofilm structure and heterogeneity of microbial communities and notably reduced autotrophic populations.

Metagenomic analysis further demonstrated that exposure to ERCA and AMR-reservoir inocula disturbed the ecological functioning of biofilm communities. This was supported by the reduction in taxonomic diversity and shifts in functional prediction, including energy and membrane metabolism (Figures S7–S10), in contrast to the increased abundance and richness of ARGs, MGEs and VFGs post-antibiotic exposure.

The observed prevalence of ARGs, such as *int1,* throughout all our pair-wise conditions (including control biofilms) questions the utility of these ARGs as discriminatory indicators of acquired AMR in freshwater environments. However, our results, in combination with previous resistome risk assessments [15,47,66], highlight *floR* and *sul1* as more selective indicators of AMR after ERCA and/or wastewater exposure. Based on microbiome profiling, further research on *Variovorax* spp., as potential indicators of increased AMR in freshwater environments, could offer valuable insights into the transmission of AMR-related elements between pathogenic organisms and environmental bacteria.

## Data Availability

The datasets presented in this study can be found in online repositories. The names of the repository/repositories and accession number(s) can be found at: https://www.ncbi.nlm.nih.gov/ (accessed on 20 May 2024), BioProject ID: PRJNA1099917.

## Supplementary Materials

The following supporting information can be downloaded at: https://www.mdpi.com/article/10.3390/antibiotics13060539/s1, Supplementary File S1, Supplementary Figures: Figure S1. Image of South Saskatchewan River showing the city of Saskatoon’s Wastewater treatment plant (WWTP) and the upstream sampling site used for water collection for the RAB microcosm system (Adapted from Google Earth). Figure S2. Structural biofilm composition. Pair-wise comparison using ANOVA post TukeyHSD between treatments based on the biofilm thickness measured from the CLSM image stained with *A. hypogaea*-FITC, *C. ensiformis*-FITC and *T. vulgaris*-TRITC. Significance is *** = 0.001, * = 0.01, * = 0.05. C = river water only, M = swine manure, W = WWTP effluent, S1: 1/10 sub-MIC, S2: 1/100 sub-MIC. Figure S3. Proportional biomass of lectin-binding specificity of the three probes used to characterize the glycoconjugate composition of the EPS matrix of biofilm communities grown under different ERCA treatments. Figure S4. Proportional biomass abundance of biofilm architecture components grown after 8-weeks under ERCA (S1: 1/10 sub-MIC, S2: 1/100 sub-MIC) and AMR-reservoir inoculant supplementation (C: river water, M: swine manure and W: WWTP effluent) treatments. Mean values are displayed with biological (*n* = 3) and technical (*n* = 5) replicates. Figure S5. Bacterial relative abundance at class taxonomic level in biofilm communities after an 8-week growth period in the presence of different ERCA or sub-MIC antibiotic treatments (1/10 and 1/100 levels) and AMR-reservoir inocula (WWTP and SM). C = river water only, S1: 1/10 sub-MIC, S2: 1/100 sub-MIC, M = manure, W = WWTP effluent. Figure S6. nMDS of Bray–Curtis similarities showing β diversity of total identified VFGs across treatments in biofilm communities. Asterisk indicates VFGs with significant abundance differences across treatments (*p* < 0.05). Ellipse shapes were defined using covariance of each group, and ellipse centroids represents the group mean (*n* = 3). VFGs: Virulence Factor Genes. Figure S7. Functional profiling related to resistome and stress-response metabolism of riverine biofilm communities under ERCA and AMR-reservoir inocula exposure. (A and B) Gene families related to AMR-elements with their relative and differential abundance (log^2^-fold change) across treatments annotated using KEGG; (C and D): Gene families related to Heavy-Metals stress response with their relative and differential abundance (log^2^-fold change) annotated using KEGG. Figure S8. Functional profiling. Number of known and unknown gene families and pathways detected using KEGG and MetaCyc databases. Figure S9. KEGG genes (*n* = 101) associated with antimicrobial resistance with significant differential abundance (*p* < 0.05) across treatments. C = river water only, S1: 1/10 sub-MIC, S2: 1/100 sub-MIC, M = manure, W = WWTP effluent. Figure S10. Pathways from MetaCyc (*n* = 72) with significant differential abundance (*p* < 0.05) across treatments. C = river water only, S1: 1/10 sub-MIC, S2: 1/100 sub-MIC, M = manure, W = WWTP effluent. Figure S11. Procrustes analysis of the microbiome and resistome. C = river water only, S1: 1/10 sub-MIC, S2: 1/100 sub-MIC, M = manure, W = WWTP effluent. Supplementary File S2, Supplementary Material; Supplementary File S3, Supplementary Tables (excel file): Table S1. α diversity indices of bacterial microbiomes across biofilm communities through different sub-MIC antibiotic and AMR-reservoir inocula (M and W) treatments. Shown are mean values with biological replicates (*n* = 3). Table S2. List of differentially abundant ARGs after antibiotic treatment exposure. Pair-wise comparison are results from DESeq2 contrast analysis. Table S3. Pairwise comparison of significant virulence factor genes after Fold change differences between treatments. Table S4. Plasmids identified in river biofilm communities after ERCA and AMR-reservoir inocula (M and W) exposure. Table S5. Concentrations of heavy metals detected across treatments exposed to sub-MIC antibiotics and AMR-reservoir inocula (M and W). Table S6. Pharmaceutical concentrations detected across river water source (RW) and AMR-reservoir inocula (M and W). References [5,7,10,13,34,66,77,82,83,84,85,86,87,88] are cited in the Supplementary Materials.
