# Supplementary material for: Environmentally Relevant Antibiotic Concentrations Exert Stronger Selection Pressure on River Biofilm Resistomes than AMR-Reservoir Effluents
Source: Antibiotics (Basel). 2024 Jun 10;13(6):539. doi: 10.3390/antibiotics13060539 (PMC11200958; doi:10.3390/antibiotics13060539)
Supplement: Supplementary file 1 [file antibiotics-13-00539-s001.zip › antibiotics-2978303-Supplementary File S1. Supp. Figures.pdf]

Environmentally relevant antimicrobial concentrations and their impact on biofilm resistomes

Gabriela Flores-Vargas [1], Jordyn Bergsveinson [2], Darren R. Korber [1,*]

[1] Food and Bioproduct Sciences, University of Saskatchewan, Saskatoon, SK, S7N 5A8, Canada
[2] Environment and Climate Change Canada, 11 Innovation Blvd., Saskatoon, SK, S7N 3H5, Canada
* Corresponding author:
drk137@mail.usask.ca

SUPLEMENTARY FILE 1, SUPPLEMENTARY FIGURES

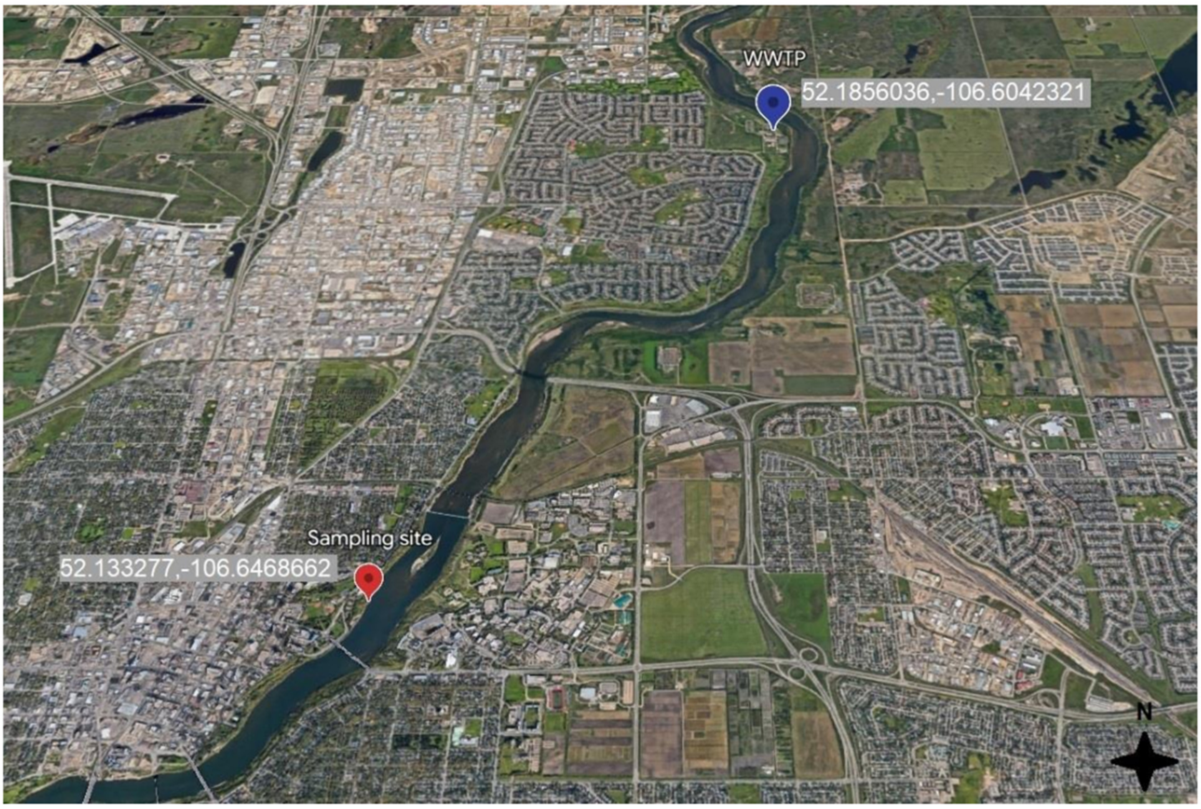

Figure S1. Image of South Saskatchewan River showing the city of Saskatoon's Wastewater treatment plant (WWTP) and the upstream sampling site used for water collection for the RAB microcosm system (Adapted from Google Earth).

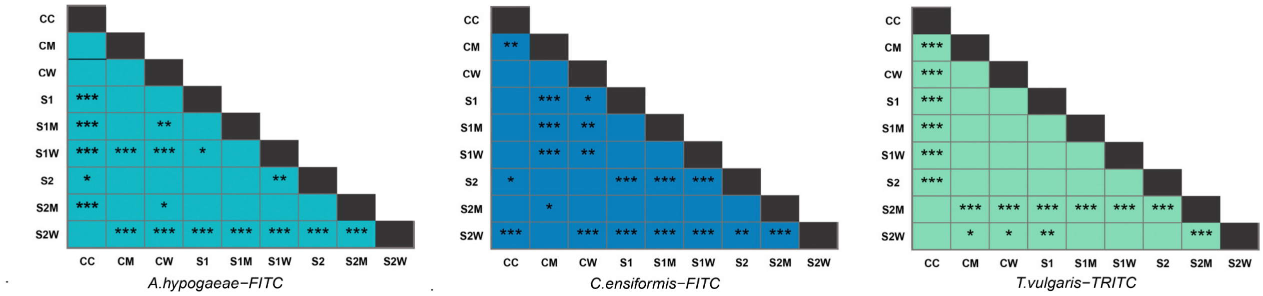

Figure S2. Structural biofilm composition. Pair-wise comparison using ANOVA post-Tukey HSD between treatments based on the biofilm thickness measured from the CLSM image stained with *A. hypogaea*-FITC, *C. ensiformis*-FITC and *T. vulgaris*-TRITC. Significance is *** = 0.001, * = 0.01, *= 0.05. C= river water only, M= swine manure, W= WWTP effluent, S1: 1/10 sub-MIC, S2: 1/100 sub-MIC.

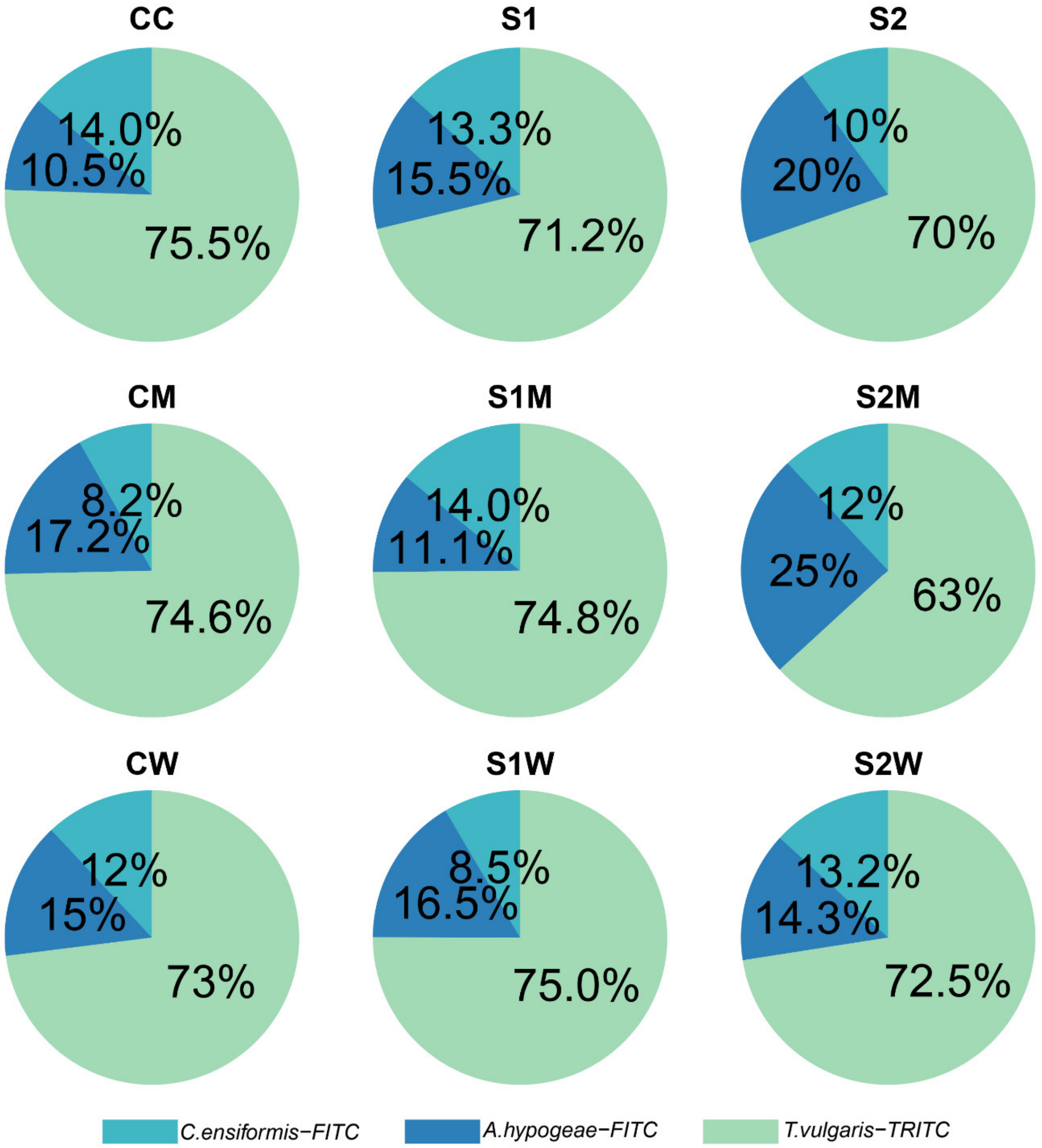

Figure S3. Proportional biomass of lectin-binding specificity of the three lectin probes used to characterize the glycoconjugate composition of the EPS matrix of biofilm communities grown under different ERCA antibiotic treatments.

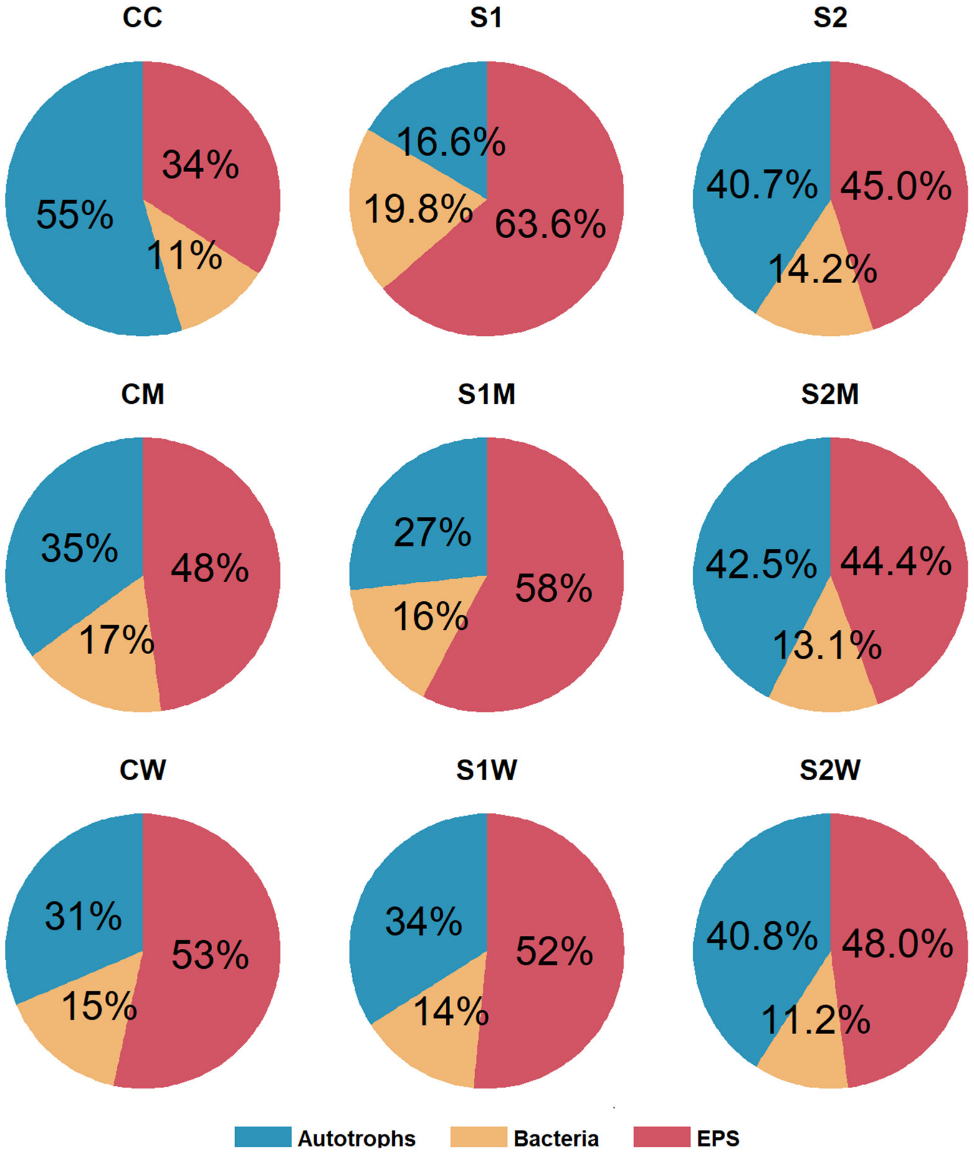

Figure S4. Proportional biomass abundance of biofilm architecture components grown after 8-weeks under ERCA (S1: 1/10 sub-MIC, S2: 1/100 sub-MIC) and AMR-reservoir inoculant supplementation (C: river water, M: swine manure and W: WWTP effluent) treatments. Mean values are displayed with biological (*n*=*3*) and technical (*n*= *5*) replicates.

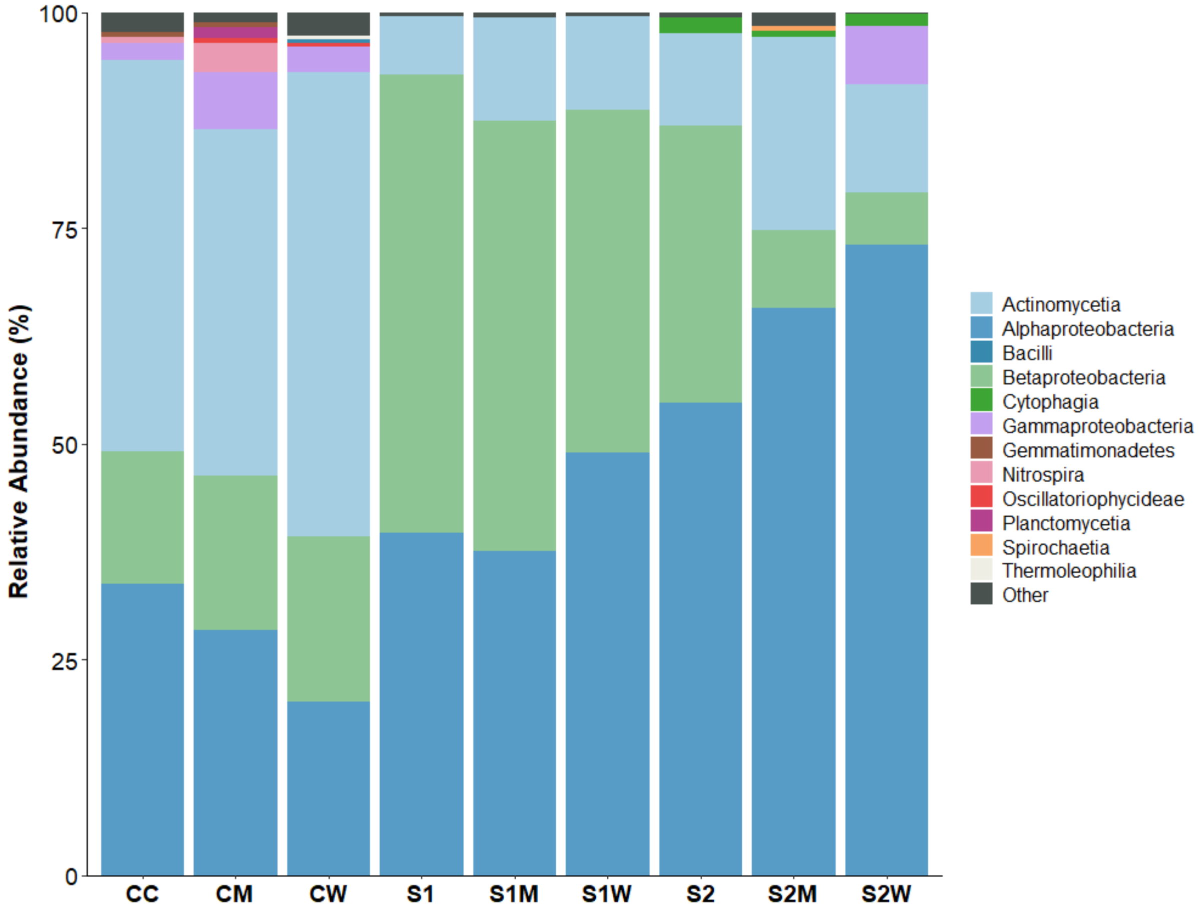

Figure S5. Bacterial relative abundance at class taxonomic level in biofilm communities after an 8-week growth period in the presence of different ERCA or sub-MIC antibiotic treatments (1/10 and 1/100 levels) and AMR-reservoir inocula (WWTP and SM). C= river water only, S1: 1/10 sub-MIC, S2: 1/100 sub-MIC, M= manure, W= WWTP effluent.

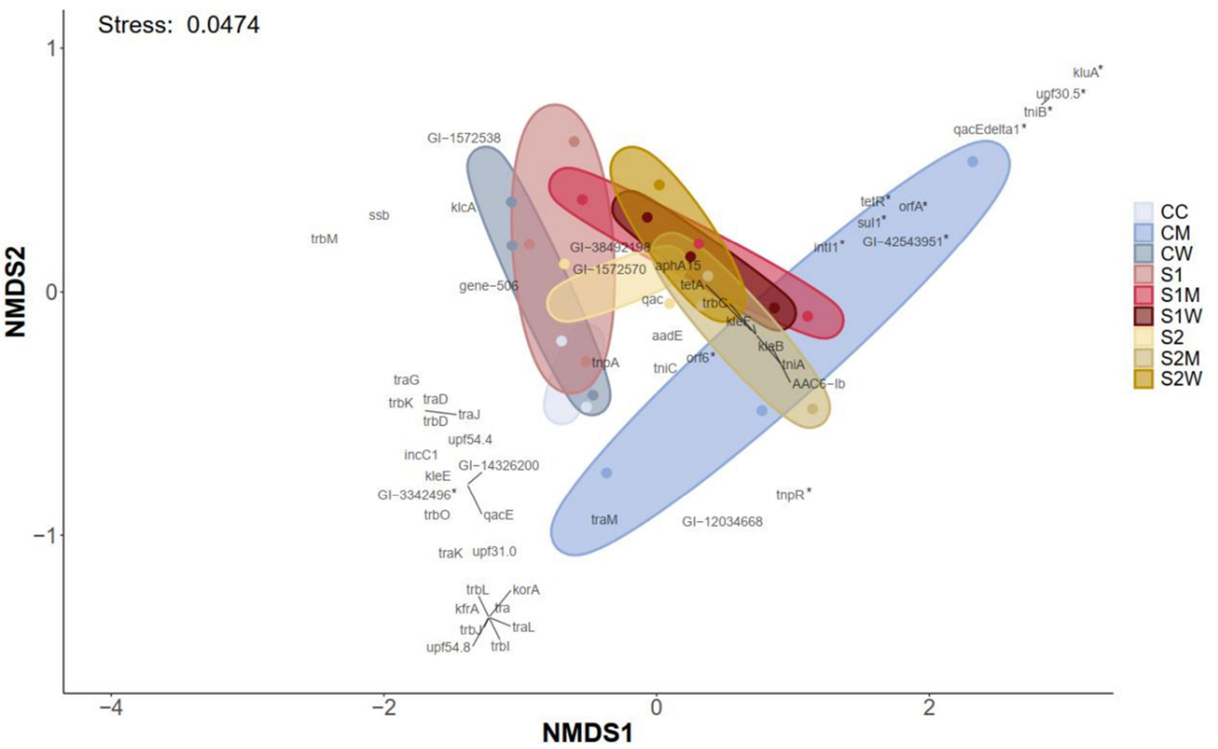

Figure S6. nMDS of Bray-Curtis similarities showing β diversity of total identified VFGs across treatments in biofilm communities. Asterisk indicates VFGs with significant abundance differences across treatments (*p*<0.05). Ellipse shapes were defined by covariance of each group, and ellipse centroids represents the group mean (*n=3*). VFGs: Virulence Factor Genes.

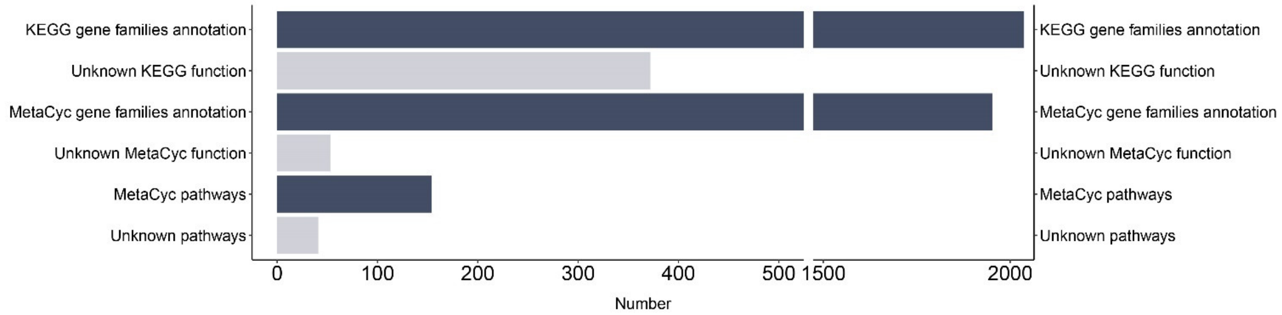

Figure S7. Functional profiling. Number of known and unknown gene families and pathways detected by KEGG and MetaCyc databases.

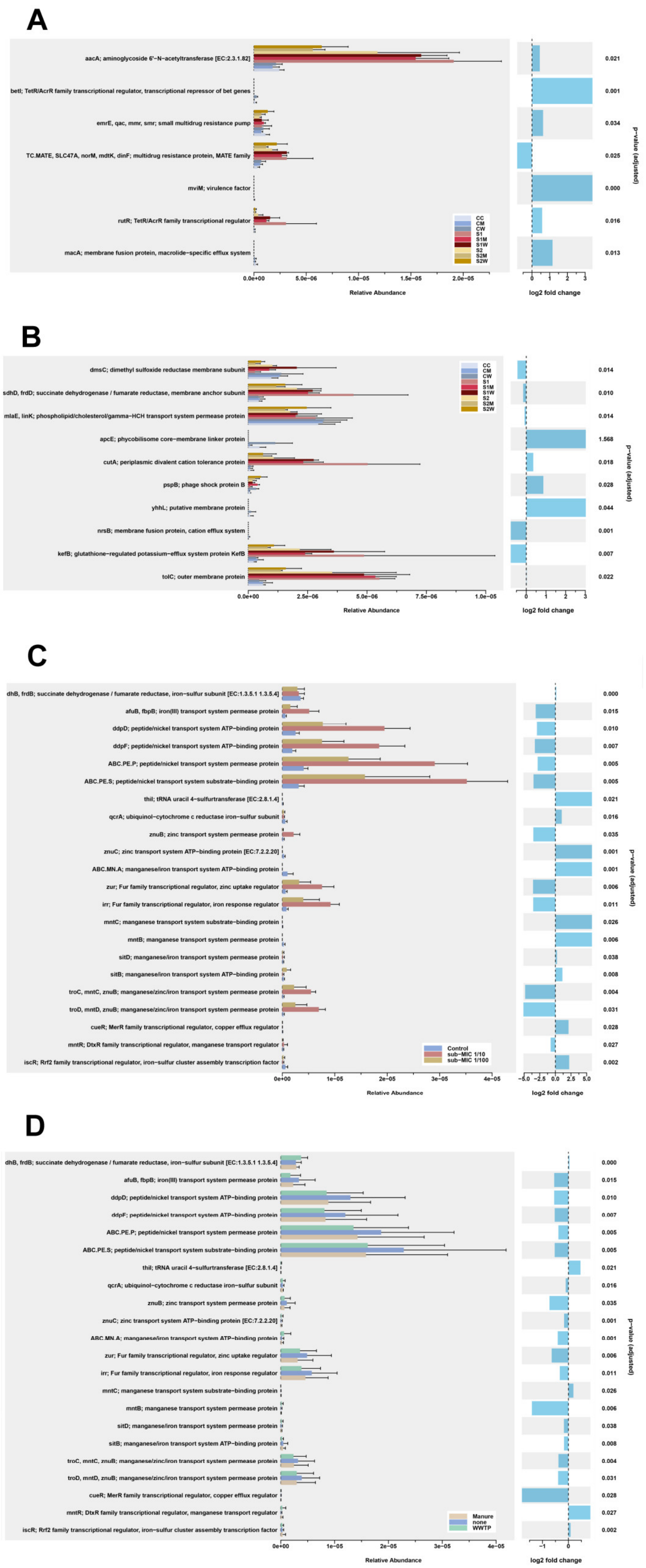
A
aacA; aminoglycoside 6'−N−acetyltransferase [EC:2.3.1.82]
betI; TetR/AcrR family transcriptional regulator, transcriptional repressor of bet genes
emrE, qac, mmr, smr; small multidrug resistance pump
TC.MATE, SLC47A, norM, mdtK, dinF; multidrug resistance protein, MATE family
mviM; virulence factor
rutR; TetR/AcrR family transcriptional regulator
macA; membrane fusion protein, macrolide−specific efflux system
CC
CM
CW
S1
S1M
S1W
S2
S2M
S2W
Relative Abundance
log2 fold change
p−value (adjusted)
0.021
0.001
0.034
0.025
0.000
0.016
0.013
B
dmsC; dimethyl sulfoxide reductase membrane subunit
sdhD, frdD; succinate dehydrogenase / fumarate reductase, membrane anchor subunit
mlaE, linK; phospholipid/cholesterol/gamma−HCH transport system permease protein
apcE; phycobilisome core−membrane linker protein
cutA; periplasmic divalent cation tolerance protein
pspB; phage shock protein B
yhhL; putative membrane protein
nrsB; membrane fusion protein, cation efflux system
kefB; glutathione−regulated potassium−efflux system protein KefB
tolC; outer membrane protein
Relative Abundance
log2 fold change
p−value (adjusted)
0.014
0.010
0.014
1.568
0.018
0.028
0.044
0.001
0.007
0.022
C
dhB, frdB; succinate dehydrogenase / fumarate reductase, iron−sulfur subunit [EC:1.3.5.1 1.3.5.4]
afuB, fbpB; iron(III) transport system permease protein
ddpD; peptide/nickel transport system ATP−binding protein
ddpF; peptide/nickel transport system ATP−binding protein
ABC.PE.P; peptide/nickel transport system permease protein
ABC.PE.S; peptide/nickel transport system substrate−binding protein
thiI; tRNA uracil 4−sulfurtransferase [EC:2.8.1.4]
qcrA; ubiquinol−cytochrome c reductase iron−sulfur subunit
znuB; zinc transport system permease protein
znuC; zinc transport system ATP−binding protein [EC:7.2.2.20]
ABC.MN.A; manganese/iron transport system ATP−binding protein
zur; Fur family transcriptional regulator, zinc uptake regulator
irr; Fur family transcriptional regulator, iron response regulator
mntC; manganese transport system substrate−binding protein
mntB; manganese transport system permease protein
sitD; manganese/iron transport system permease protein
sitB; manganese/iron transport system ATP−binding protein
troC, mntC, znuB; manganese/zinc/iron transport system permease protein
troD, mntD, znuB; manganese/zinc/iron transport system permease protein
cueR; MerR family transcriptional regulator, copper efflux regulator
mntR; DtxR family transcriptional regulator, manganese transport regulator
iscR; Rrf2 family transcriptional regulator, iron−sulfur cluster assembly transcription factor
Control
sub−MIC 1/10
sub−MIC 1/100
Relative Abundance
log2 fold change
p−value (adjusted)
D
Manure
none
WWTP
Relative Abundance
log2 fold change
p−value (adjusted)

Figure S8. Functional profiling related to resistome and stress-response metabolism of riverine biofilm communities under ERCA and AMR-reservoir inocula exposure. A and B) Gene families related to AMR-elements with their relative and differential abundance (log^2-fold change) across treatments annotated by KEGG; C and D): Gene families related to Heavy-Metals stress response with their relative and differential abundance (log^2-fold change) annotated by KEGG.

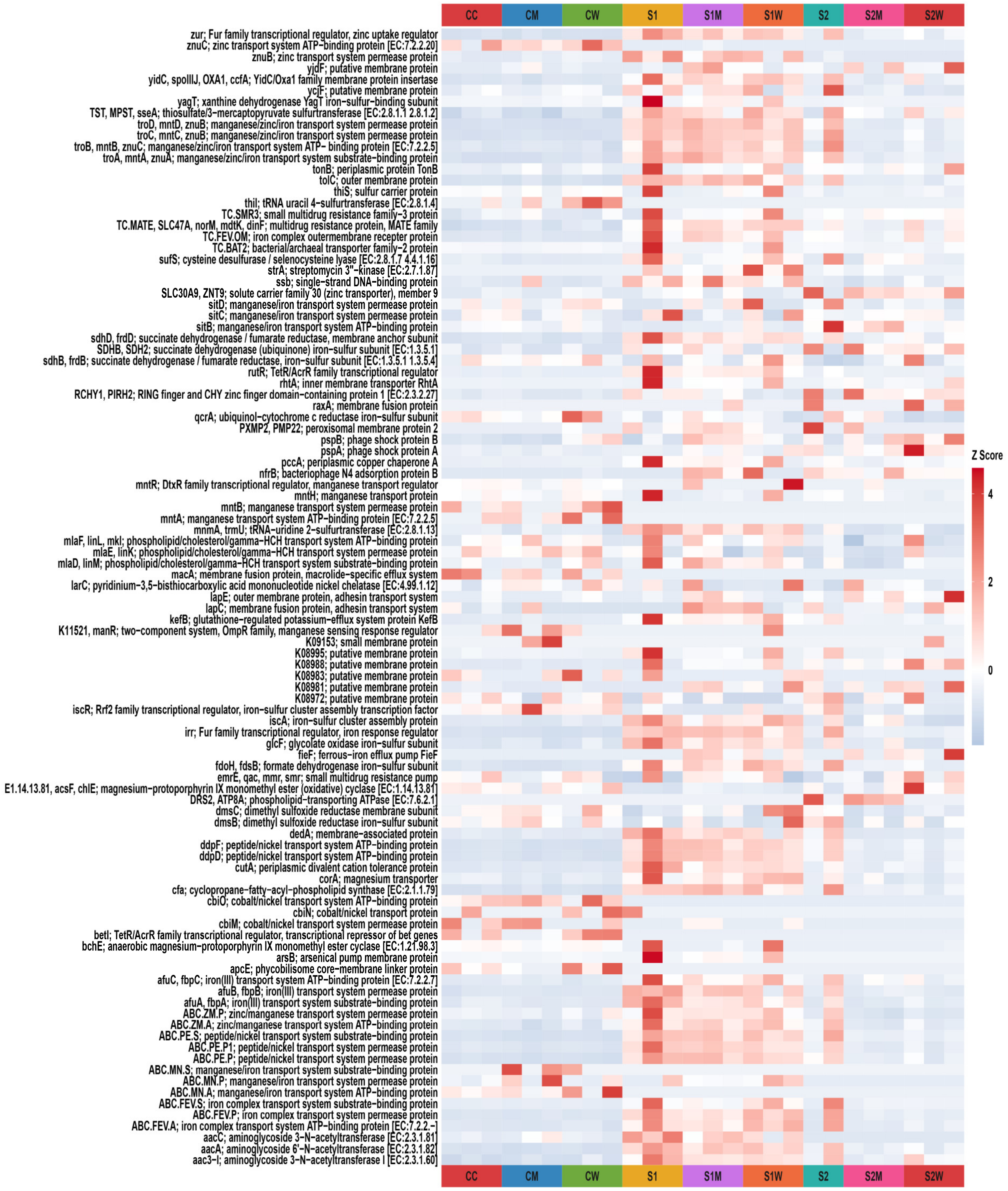

Figure S9. KEGG genes (n = 101) associated with antimicrobial resistance and having significant differential abundance ($p<0.05$) across treatments. C= river water only, S1: 1/10 sub-MIC, S2: 1/100 sub-MIC, M= manure, W= WWTP effluent.

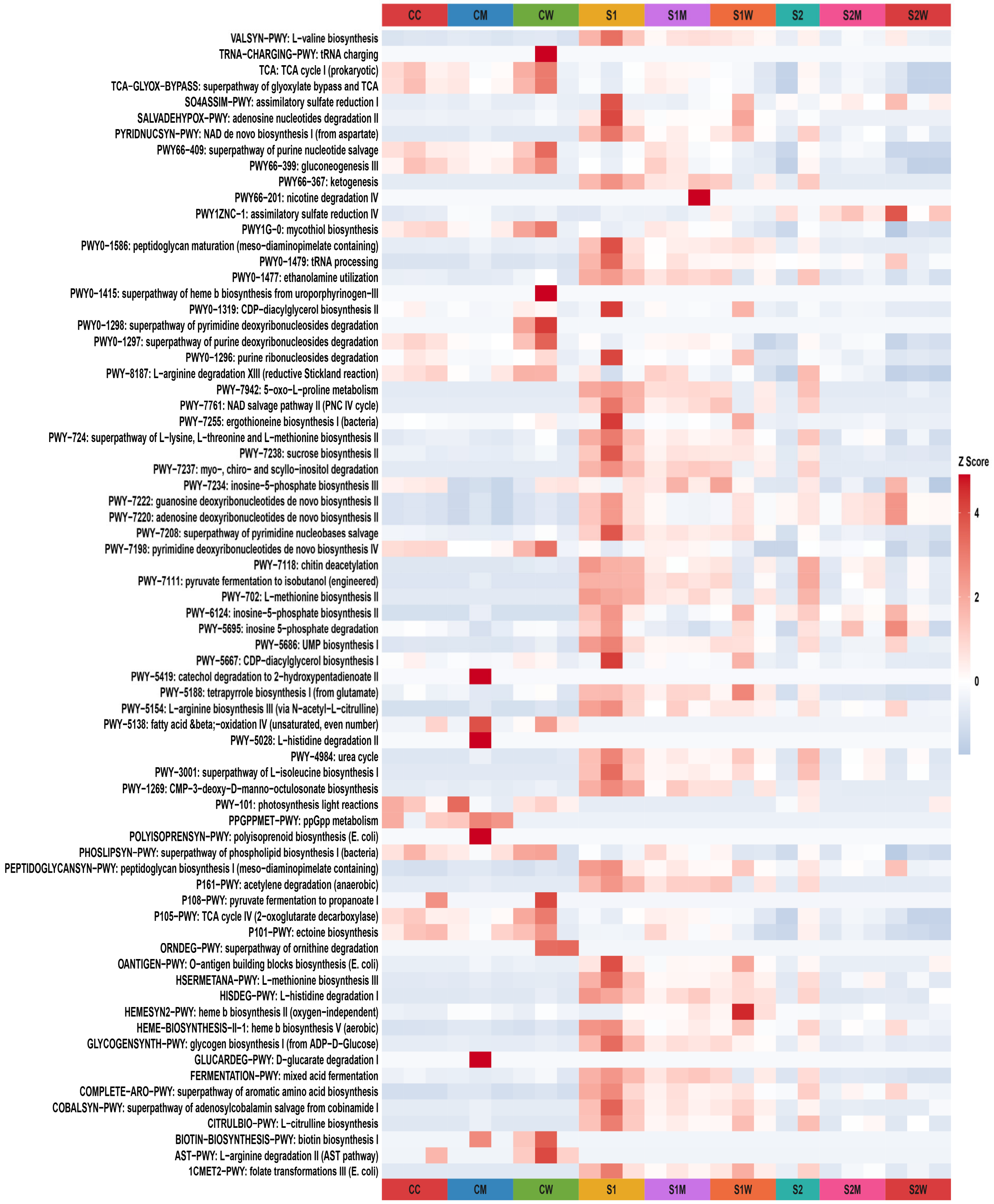

Figure S10. Pathways from MetaCyc (n=72) with significant differential abundance (p < 0.05) across treatments. C= river water only, S1: 1/10 sub-MIC, S2: 1/100 sub-MIC, M= manure, W= WWTP effluent.

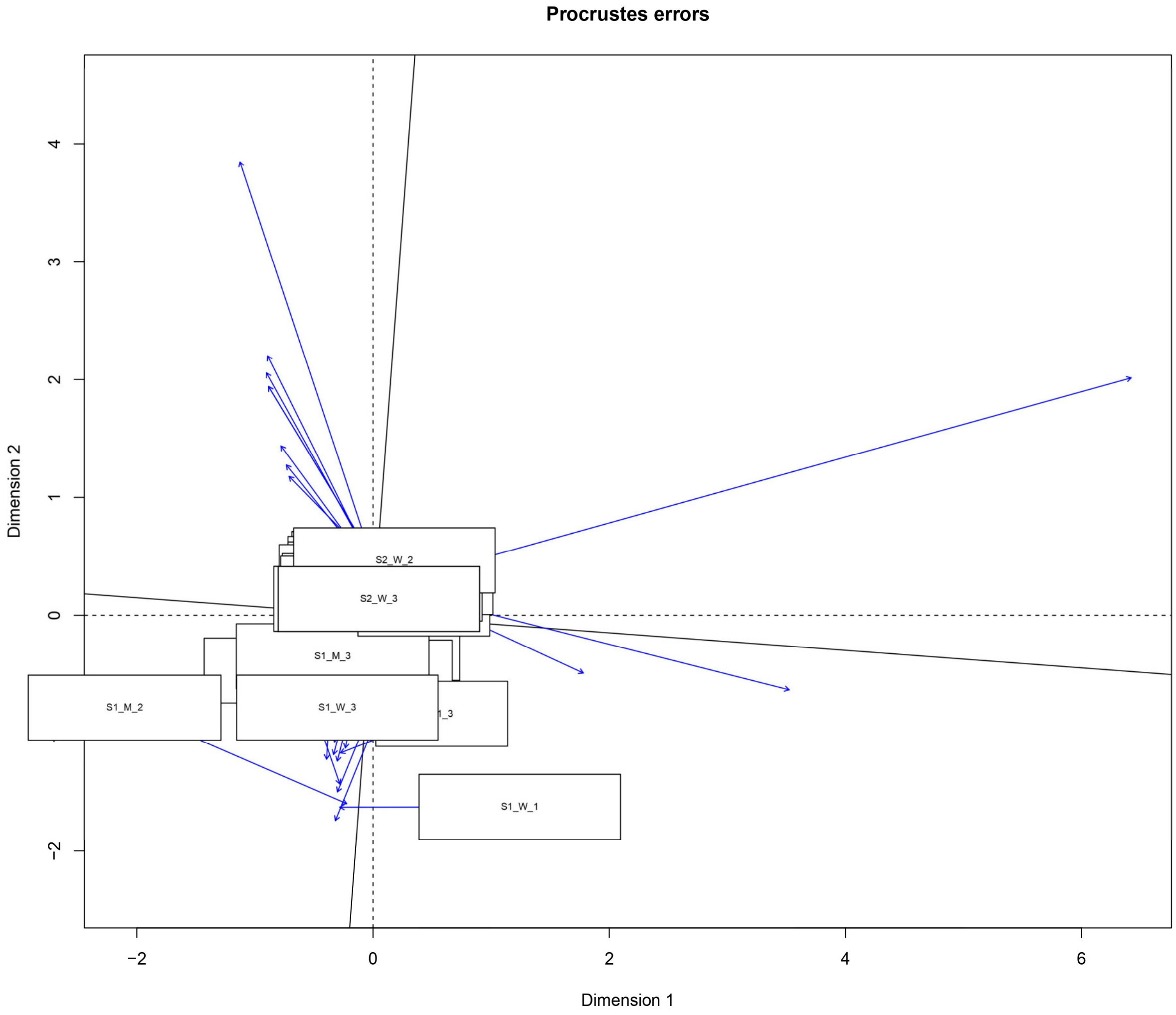

Figure S11. Procrustes analysis of the microbiome and resistome. C= river water only, S1: 1/10 sub-MIC, S2: 1/100 sub-MIC, M= manure, W= WWTP effluent.
