# Supplementary material for: Environmentally Relevant Antibiotic Concentrations Exert Stronger Selection Pressure on River Biofilm Resistomes than AMR-Reservoir Effluents
Source: Antibiotics (Basel). 2024 Jun 10;13(6):539. doi: 10.3390/antibiotics13060539 (PMC11200958; doi:10.3390/antibiotics13060539)
Supplement: Supplementary file 1 [file antibiotics-13-00539-s001.zip › antibiotics-2978303-Supplementary File S2. Supp. Material&Methods.pdf]

Environmentally relevant antibiotic concentrations exert stronger selection pressure on river biofilm resistomes than AMR-reservoir effluents

Gabriela Flores-Vargas [1], Jordyn Bergsveinson [2], Darren R. Korber [1,*]

[1] Food and Bioproduct Sciences, University of Saskatchewan, Saskatoon, SK, S7N 5A8, Canada
[2] Environment and Climate Change Canada, 11 Innovation Blvd., Saskatoon, SK, S7N 3H5, Canada
* Corresponding author:
drk137@mail.usask.ca

SUPLEMENTARY FILE 2, SUPPLEMENTARY MATERIAL

1. Materials and Methods

1.1. Microcosm experimental design, ERCA exposure

Antibiotic	Drug class	MIC *	sub-MIC 1/10	sub-MIC 1/100
Ciprofloxacin	Fluoroquinolone	0.5 mg/L	50 µg/L	5 µg/L
Oxytetracycline	Tetracycline	125 mg/L	12,500 µg/L	1250 µg/L
Streptomycin	Aminoglycoside	512 mg/L	51,200 µg/L	5120 µg/L

* MIC values for the biofilm communities were determined based on breakpoint reports of known pathogen isolates from EUCAST (European Committee on Antimicrobial Testing) and CLSI (Clinical Laboratory Standards Institute).

1.2. Antimicrobial and heavy metal quantification

Antimicrobial and heavy metal concentrations of the river water (RW) and W and M AMR-reservoir inputs were analyzed. Total concentrations of 23 heavy metals (Al, As, B, Ba, Cd, Cr, CN-, Co, Cu, Fe, Hg, Mn, Mo, Ni, Pb, Sb, Se, Sn, Sr, Ti, U, V, Zn) were measured from 1 L of each treatment by SRC (Saskatchewan Research Council) Environmental Analytical Laboratories. Screening for a 25-drug antimicrobial panel of clinical importance, including ciprofloxacin, was conducted at Innotech-Alberta Pharmaceutical Analytical Services from 500 mL of all inputs.

1.3. Metagenomic functional profiling

Prediction of functional of genes associated with AMR and stress-response pathways were identified through the HUMAnN v.3.1.1 pipeline [1]. The same input file per sample as in CosmosID (subsampled-trimmed paired-end reads merged into a single file) were mapped against the ChocoPhlAn and the full UniRef (version: uniref90_201901b) database. Default

parameters were used and pathway abundances of detected gene families were calculated using the MetaCyc database [2]. For output from the KEGG metabolic database [3], the "humann_regroup_table" with the '–groups uniref90_ko' module was used. The abundance of each pathway (path_abundance.tsv file) of all samples were merged into a single table with "humann_join_tables" option. The list of each identified pathway was represented in reads per kilobase (RPK) units with abundances grouped in stratifications consisting of pathways assigned to bacterial genera. After filtering out unidentified functional pathways, a pathway abundance table was used for subsequent multivariate and statistical analyses.

2. Results and Discussion

2.1. Trace metal and antibiotic analysis

Given that the occurrence of heavy or trace metals is frequently associated with AMR [4], the trace concentrations of metals was quantified within the South Saskatchewan River and the two AMR-reservoir sources (M and W) used in our study. Results revealed that levels of Hg, Sr, and U were higher in W compared to M and river water (Table S5), though there was interesting peak, Ultimately, no significant differences in metal levels were recorded across AMR-reservoir inocula or sub-MIC antibiotic conditions relative to controls. Cu and Zn were detected in all treatments and AMR-reservoir conditions (M, W), although levels were higher in W. The majority of the other 23 screened heavy metals were below the limit of detection (<LOD), which highlights one of the challenges in quantifying pollutants in natural environments, as antibiotics and metals are often present at very low concentrations (ng/L–µg/L) [5,6].

From the 25 screened pharmaceuticals, 14 antimicrobials were detected over the limit of detection in the river water (RW), M and W (Table S6) treatments, while all other pharmaceuticals were <LOD, yet the highest concentrations were observed in M (10.8 – 17.9 µg/L) with four pharmaceuticals: acetaminophen, sulfadoxine, sulfamethazine and sulfathiazole. The three sulfa-class antimicrobials (sulfadoxine, sulfamethazine and sulfathiazole) were also detected in M samples. Ciprofloxacin was detected in RW and W samples, implying that traces of this antibiotic persist in downstream environments after urban usage, similar to records from rivers across North America [6]. It has been shown that ciprofloxacin concentrations equal or higher to 11.5 µg/L in river biofilms influenced by WWTP discharge favors the enrichment of existing ciprofloxacin-resistant mutants, and have the potential to select for resistant mutants from non-resistant populations [7]. Thus, while it is important to confirm detection of specific compounds present in environmental samples, it is noteworthy that while they may be present at extremely dilute concentrations, antimicrobials have the potential to induce unanticipated but related effects on non-target organisms, promote the development of other mechanisms of tolerance, and/or exert yet unknown actions when present at trace concentrations [8,9].

2.2. Functional analysis related to AMR and stress response

Gene families and metabolic pathways of all samples were annotated to identify significant changes in the abundance of genes associated with the resistome and stress response. Annotations performed by the KEGG and MetaCyc database identified a total of 2,037 and 1,952

gene families, respectively (Figure S7). Differential RPK abundance revealed 1,620 gene families annotated by KEGG with significant changes across treatments (*p*<0.05; Figure S8), of which 101 genes associated with AMR elements were used for visualization and analysis (Figure S9). Moreover, 72 pathways detected through MetaCyc yielded significant differential RPK abundance across treatments (*p*<0.05, Figure S10). The differences in annotations highlight the importance of metagenomic pipeline selection and/or comparison when analyzing environmental samples, as previously noted [10,11].

AMR-related gene elements annotated by KEGG were consistent with the resistome composition previously described above. For example, the aminoglycoside 6'-N-acetyltransferase family (*aacA*) showed significantly increased abundance in antibiotic treated samples (S1, S1M, S1W, S2, S2M and S2W) relative to controls, particularly in the 1/10 sub-MIC condition (S1) (Figure S8). Similarly, gene families related to cell-wall and cell-membrane metabolism showed differential abundances between untreated and treated biofilms (Figure S8). This observation is further supported by MetaCyc results, which annotated three significant membrane metabolism pathways related to peptidoglycan and phospholipid biosynthesis (Figure S8B). Alterations to the membrane metabolism and synthesis has been associated with a reduction of bacterial biofilm formation after antibiotic exposure [12].

From the 154 total metabolic pathways annotated by MetaCyc, 114 pathways revealed significant differential abundances across our biofilms and treatments. In general, pathways associated with amino acids biosynthesis of L-serine, glycine, L-isoleucine, and aromatics, as well as *de novo* nucleic acid biosynthesis or salvage (adenosine, pyrimidine, purine, guanosine, and diphosphate), were significantly increased in antibiotic treated samples relative to controls. However, pathways of specific amino acid biosynthesis (L-aspartate, L-asparagine, and cysteine) and nucleic acid salvage (pyrimidine and purine) biosynthesis were observed with significantly decreased RPK over similar condition comparisons.

Interestingly, several gene families associated with metal stress response showed higher abundance in samples treated with 1/10 sub-MIC antibiotics relative to control samples, such as iron response regulator (*irr*), peptide/nickel transport system ATP-binding protein, (*ddpD; ddpF*), and peptide/nickel transport system substrate–binding protein (Figure S8C-D). Despite the detected concentrations of these metals in the samples being minimal, the relative abundance of these genes being higher in samples exposed to 1/10 sub-MIC antibiotics supports the assertion that the presence of antibiotic selective pressure favours the maintenance of metal resistance genes [13,14].

REFERENCES

1. Beghini, F.; McIver, L.J.; Blanco-Míguez, A.; Dubois, L.; Asnicar, F.; Maharjan, S.; Mailyan, A.; Manghi, P.; Scholz, M.; Thomas, A.M.; et al. Integrating Taxonomic, Functional, and Strain-Level Profiling of Diverse Microbial Communities with BioBakery 3. *Elife* **2021**, *10*, e65088, doi:10.7554/eLife.65088.

2. Kanehisa, M.; Goto, S.; Sato, Y.; Kawashima, M.; Furumichi, M.; Tanabe, M. Data, Information, Knowledge and Principle: Back to Metabolism in KEGG. *Nucleic Acids Res.* **2013**, *42*, D199–D205, doi:10.1093/nar/gkt1076.

3. Caspi, R.; Billington, R.; Fulcher, C.A.; Keseler, I.M.; Kothari, A.; Krummenacker, M.; Latendresse, M.; Midford, P.E.; Ong, Q.; Ong, W.K.; et al. The MetaCyc Database of Metabolic Pathways and Enzymes. *Nucleic Acids Res.* **2018**, *46*, D633–D639, doi:10.1093/nar/gkx935.

4. Xue, J.; Wu, J.; Hu, Y.; Sha, C.; Yao, S.; Li, P.; Lin, K.; Cui, C. Occurrence of Heavy Metals, Antibiotics, and Antibiotic Resistance Genes in Different Kinds of Land-Applied Manure in China. *Environ. Sci. Pollut. Res.* **2021**, *28*, 40011–40021, doi:10.1007/s11356-021-13307-9.

5. Chow, L.K.M.; Ghaly, T.M.; Gillings, M.R. A Survey of Sub-Inhibitory Concentrations of Antibiotics in the Environment. *J. Environ. Sci.* **2021**, *99*, 21–27, doi:https://doi.org/10.1016/j.jes.2020.05.030.

6. Wilkinson, J.L.; Boxall, A.B.A.; Kolpin, D.W.; Leung, K.M.Y.; Lai, R.W.S.; Galbán-Malagón, C.; Adell, A.D.; Mondon, J.; Metian, M.; Marchant, R.A.; et al. Pharmaceutical Pollution of the World's Rivers. *Proc. Natl. Acad. Sci.* **2022**, *119*, e2113947119, doi:10.1073/pnas.2113947119.

7. Matviichuk, O.; Mondamert, L.; Geffroy, C.; Dagot, C.; Labanowski, J. Life in an Unsuspected Antibiotics World: River Biofilms. *Water Res.* **2023**, *231*, doi:10.1016/j.watres.2023.119611.

8. Bengtsson-Palme, J.; Larsson, D.G.J. Concentrations of Antibiotics Predicted to Select for Resistant Bacteria: Proposed Limits for Environmental Regulation. *Environ. Int.* **2016**, *86*, 140–149, doi:10.1016/j.envint.2015.10.015.

9. Tarek, M.H.; Garner, E. A Proposed Framework for the Identification of Indicator Genes for Monitoring Antibiotic Resistance in Wastewater: Insights from Metagenomic Sequencing. *Sci. Total Environ.* **2023**, *854*, 158698, doi:10.1016/j.scitotenv.2022.158698.

10. Bharti, R.; Grimm, D.G. Current Challenges and Best-Practice Protocols for Microbiome Analysis. *Brief. Bioinform.* **2021**, *22*, 178–193, doi:10.1093/bib/bbz155.

11. Flores-Vargas, G.; Korber, D.R.; Bergsveinson, J. Sub-MIC Antibiotics Influence the Microbiome, Resistome and Structure of Riverine Biofilm Communities. *Front. Microbiol.* 2023, *14*. 1194952..

12. Hall, C.W.; Mah, T.-F. Molecular Mechanisms of Biofilm-Based Antibiotic Resistance and Tolerance in Pathogenic Bacteria. *FEMS Microbiol. Rev.* **2017**, *41*, 276–301, doi:10.1093/femsre/fux010.

13. Zhang, M.; Liu, Y.S.; Zhao, J.L.; Liu, W.R.; He, L.Y.; Zhang, J.N.; Chen, J.; He, L.K.; Zhang, Q.Q.; Ying, G.G. Occurrence, Fate and Mass Loadings of Antibiotics in Two Swine Wastewater Treatment Systems. *Sci. Total Environ.* **2018**, *639*, 1421–1431, doi:10.1016/j.scitotenv.2018.05.230.
14. Liu, C.; Li, X.; Zheng, S.; Kai, Z.; Jin, T.; Shi, R.; Huang, H.; Zheng, X. Effects of Wastewater Treatment and Manure Application on the Dissemination of Antimicrobial Resistance around Swine Feedlots. *J. Clean. Prod.* **2021**, *280*, 123794, doi:https://doi.org/10.1016/j.jclepro.2020.123794.
